# The effectiveness of interventions designed to increase the uptake of clinical practice guidelines and best practices among musculoskeletal professionals: a systematic review

**DOI:** 10.1186/s12913-018-3253-0

**Published:** 2018-06-08

**Authors:** Fadi M. AL Zoubi, Anita Menon, Nancy E. Mayo, André E. Bussières

**Affiliations:** 1 School of Physical and Occupational Therapy, Faculty of Medicine, McGill University, 3630 Promenade Sir-William-Osler, Hosmer House, 16 Room 205, Montreal, QC, H3G 1Y5 Canada; 2 Centre de recherche interdisciplinaire en réadaptation (CRIR), Montréal, QC, Canada; 3 Département chiropratique, Université du Québec à Trois-Rivières, Trois-Rivières, QC, Canada

**Keywords:** Knowledge translation, Systematic review, Implementation science, Musculoskeletal disorders, Musculoskeletal professionals

## Abstract

**Background:**

The objective of this systematic review was to summarize and evaluate evidence about the effectiveness of knowledge translation (KT) interventions to improve the uptake and application of clinical practice guidelines and best practices for a wide range of musculoskeletal (MSK) disorders and health care professionals.

**Methods:**

A search for relevant randomized controlled trials (RCTs) published in English was conducted in MEDLINE (Ovid interface), EMBASE, CINAHL, and CENTRAL (Cochrane library). Two independent reviewers selected studies, assessed risk of bias, and extracted data. All MSK disorders were included except MSK injuries, fractures, trauma, or inflammatory disorders.

**Results:**

A total of 7904 citations yielded 11 eligible RCTs. The targeted MSK disorders included: low back pain (*n* = 5), neck pain (*n* = 2), whiplash (1), spinal disorders (*n* = 1), and osteoarthritis of the hip and knee (n = 2). Studies primarily involved physiotherapists, chiropractors, and a mix of physiotherapists, chiropractors and osteopaths. Results were reported using effect sizes (Cohen’s *d*). Interactive educational meetings were the most commonly used KT strategy. For professional outcomes, 3 studies using *single*-*component* interventions had a small effect (*d* ranges from 0.14 to 0.28) and 7 studies used *multifaceted* interventions (3 were effective (*d* ranges from 0.824 to 2.27). For patient outcomes, 4 studies were ineffective (*d* ranges from 0.06 to 0.31). The majority of the included RCTs had moderate-to-high risk of bias. About half of the studies used theory-based interventions, but the elements of the interventions and theoretical frameworks were often poorly described. Furthermore, there were no comparable outcome measures to evaluate the impact of the interventions on a similar scale.

**Conclusions:**

The findings suggested that *multifaceted* educational KT interventions appear to be effective for improving professional outcomes, although effects were inconsistent. The KT strategies were generally not effective on patient outcomes. In general, studies were of low quality, interventions were poorly described, and only half had theoretical underpinning. Researchers are encouraged to use validated professional and patient outcomes.

**Electronic supplementary material:**

The online version of this article (10.1186/s12913-018-3253-0) contains supplementary material, which is available to authorized users.

## Background

Musculoskeletal (MSK) disorders are the second leading cause of disability globally and account for 21.3% of the total years lived with disability [1, 2]. About half (49.6%) of the total MSK disability stems from low back pain (LBP), followed by neck pain (20.1%), non-spinal MSK disorders (17.3%), osteoarthritis (10.5%), rheumatoid arthritis (2.3%), and gout (0.1%) [3]. Among people with MSK disorders, pain is the most common reason to consult health care providers in primary care [4–7]. In addition to the large impact on individuals, MSK conditions are associated with a massive social and economic burden to society [8–12].

Despite available evidence-based guidelines on the management of patients with MSK disorders [13–15], numerous professional barriers (e.g., lack of awareness, skills, self-capacity and motivation) impede the routine application of guideline recommendations in clinical practice [16, 17]. The field of knowledge translation (KT) has produced a plethora of tools and methods to address these barriers and enhance the uptake of guidelines by clinicians. The field of KT is focused on closing the gap between what is known to work best and what is routinely done in practice [18]. The closure of this gap can be achieved through developing and implementing KT interventions [19].

Most systematic reviews on the effectiveness of KT interventions to increase the uptake of clinical practice guidelines or best practices have targeted physicians [20–24] and nurses [25–28]. More recently, five systematic reviews focused on allied health professionals’ uptake of guidelines [19, 29–32]. Two of the reviews [29, 30] concluded that multifaceted KT interventions among physiotherapists can improve professional outcomes. However, one review failed to show improvement of patient outcomes [30]. In 2012, Scott et al. [19] conducted a review targeting five allied health professions (dietetics, occupational therapy, pharmacy, physiotherapy, and speech-language pathology). The search was later updated by Jones et al. (2015) [31] targeting three allied health professions (occupational therapists, physical therapists, and speech-language pathologists). These two reviews suggested that generally the studies were of poor methodological quality which precluded any decision about the effective KT intervention. A fifth review [32] evaluating the effectiveness of KT interventions to change clinical practice of physiotherapists managing common MSK disorders found equivocal effects for professional and patient outcomes.

To date, no single review has targeted other MSK professionals working in orthopedics, rheumatology, manual therapy, chiropractic, osteopathy, athletic therapy, sports medicine, acupuncture, among other areas. Thus, the goal of this review was to summarize and evaluate evidence about the effectiveness of KT interventions to improve the uptake and application of clinical practice guidelines and best practices for MSK disorders among MSK professionals.

This review addressed the following question: among MSK professionals, to what extent do KT interventions impact on (i) uptake of clinical practice guidelines or best practices for MSK disorders, and (ii) patient outcomes? For the purpose of this review, MSK professionals are health care providers whose nature and scope of practice primarily involves managing MSK disorders.

## Methods

This review followed the recommendations of Cochrane Handbook for Systematic Reviews of Interventions Version 5.1.0 [33].

### Search strategy

The following electronic databases were searched from inception to August 10th, 2016: MEDLINE (Ovid interface), EMBASE, CINAHL, and CENTRAL (Cochrane library). The search strategy (Additional file 1) was developed in conjunction with an expert health sciences librarian at McGill University. The search strategy was built using four key terms, MSK disorders, MSK health care professionals, KT interventions, and a filter for RCTs. The search strategy was developed in MEDLINE and translated into the other databases using the appropriate MESH terms as applicable. A validated search strategy with a filter for KT interventions [34] was used with some modifications (e.g. removing keywords such as nurse, pharmacist, and general practitioner) to fit our review question.

### Inclusion and exclusion criteria

#### Control group studies

Only randomized controlled trials (RCTs) were included in this review as they are considered the gold standard for examining the impact (or causal relationship) of an intervention and providing unbiased estimates of the intervention effects for the outcomes of interest [35, 36]. Furthermore, trials published in English were eligible for this review; it was deemed that this restriction is unlikely to bias the findings since most RCTs are published in English [37–41]. Non-RCTs, uncontrolled studies, and observational studies were excluded. Protocols, commentaries, conference proceedings, and reviews were also excluded.

#### Types of participants

##### MSK professionals

For the purpose of this review, MSK professionals included: physiotherapists, occupational therapists, manual therapists, chiropractors, athletic therapists, sport therapists, sport physicians, massage therapists, osteopaths, osteopathic physicians, exercise physiologists, kinesiologists, physiatrists, orthopedists, podiatrists, orthopedic surgeons, and acupuncturists. Studies targeting students were excluded.

##### Patients

All types of MSK conditions were included, except patients with MSK injuries, fractures, trauma, or inflammatory disorders (such as rheumatoid arthritis and ankylosing spondylitis). Furthermore, pregnant or pediatric-based MSK disorders were excluded.

#### Types of interventions

Studies that primarily aimed to evaluate all types of KT interventions (professional, financial, organizational, and regulatory) designed to improve the uptake and use of clinical practice guidelines or best evidence targeting MSK professionals working with MSK patients, were included. Studies had to mention the source and content of the evidence that had been transferred with the appropriate references. Studies with patient-directed interventions without the involvement of MSK professionals in the intervention were excluded. Pharmacological-based or surgical-based studies were excluded as well.

#### Types of outcome measures

We included studies with any primary outcome relevant to the objective of this review that assessed change, whether quantitatively or qualitatively, at the professional/process level (such as change in practice or behavior, knowledge, skills, self-efficacy) or patient level (such as change in knowledge, health status, pain, disability). Outcomes relevant to economic level were excluded. Table 1 provides a summary of the inclusion/exclusion criteria for this review.

**Table 1:** Eligibility criteria

Characteristics	Included	Excluded
Types of studies	RCTs	Non-RCTs, uncontrolled studies, and observational studies.
	Published in English language	Protocols, commentaries, conference proceedings, and reviews
Types of participants	MSK professionals: (PTs, OTs, manual therapists, DCs, athletic therapists, sport therapists, sport physicians, massage therapists, osteopaths, osteopathic physicians, exercise physiologists, kinesiologists, physiatrists, orthopedists, Doctors of Podiatric Medicine, orthopedic surgeons, and acupuncturists) Patients: All types of MSK conditions	General practitioners, nurses. Studies targeted students Pregnant Non-adult based MSK disorders MSK injuries, fractures, trauma, or inflammatory disorder (such as rheumatoid arthritis and ankylosing spondylitis).
Types of interventions	Studies had to mention the source and content of the evidence that had been transferred with the relevant references.	Studies with patient-directed intervention without the involvement of MSK professionals in the designed intervention
	All types of KT intervention/s (professional, financial, organizational, and regulatory)	Studies that investigated the assessment or treatment on specific MSK disorders Pharmacological-based, or surgical-based studies
Types of outcome measures	Quantitative and qualitative professional/process level or patient level outcome measure	Economic level outcomes

*RCTs* Randomized controlled trials; *PTs* Physical therapists; *OTs* Occupational therapists; *MSK* Musculoskeletal; *KT* Knowledge intervention; *DCs* Doctors of chiropractic

#### Study selection and data abstraction

All citations retrieved from literature searches were imported into EndNote and duplicates were removed. After a calibration exercise, the selection criteria were applied independently by two reviewers (FZ, AM) on all citations’ titles and abstracts. These criteria were then applied to the full text of eligible studies. One reviewer (FZ) undertook the data extraction of the included studies using a modified EPOC Data Collection Checklist [42], and the another reviewer reviewed the extracted data. At all steps, disagreements were resolved by discussion between the reviewers; if no agreement was reached, a third reviewer (AB) was asked to resolve differences. Articles deemed unsuitable for inclusion are listed in the additional file 2, along with the reason for their exclusion.

#### Quality assessment

Two reviewers independently assessed the risk of bias of the included studies using the criteria developed by the Cochrane Handbook for Risk of Bias Assessment (version 5.1.0) [33], which included random sequence generation, allocation concealment, blinding of participants and personnel, blinding of outcome assessment, incomplete outcome data, selective outcome reporting, and other potential sources of bias. Studies deemed as poor quality with a high risk for bias were included in this analysis. RevMan 5.3 software [43] was used to provide a graphical representation for risk of bias.

#### Data analysis

For both professionals and patients, studies were categorized according to their outcomes. A meta-analysis was not conducted due to the substantial heterogeneity (divergence) in the methodological quality, type of KT interventions, outcomes measures, and the type of MSK disorder under consideration across studies. Therefore, the findings were synthesized in a narrative manner. Effect sizes were calculated for both professional and patient outcomes using the differences in means between intervention and control groups over time, divided by pooled standard deviation [44]. The common rule of thumb was used for interpreting effect sizes (Cohen’s *d*) in the following manner: small (*d* = 0.2), medium (*d* = 0.5), and large (*d* = 0.8) [45].

## Results

### Search results and characteristics of the included studies

**Fig. 1:**
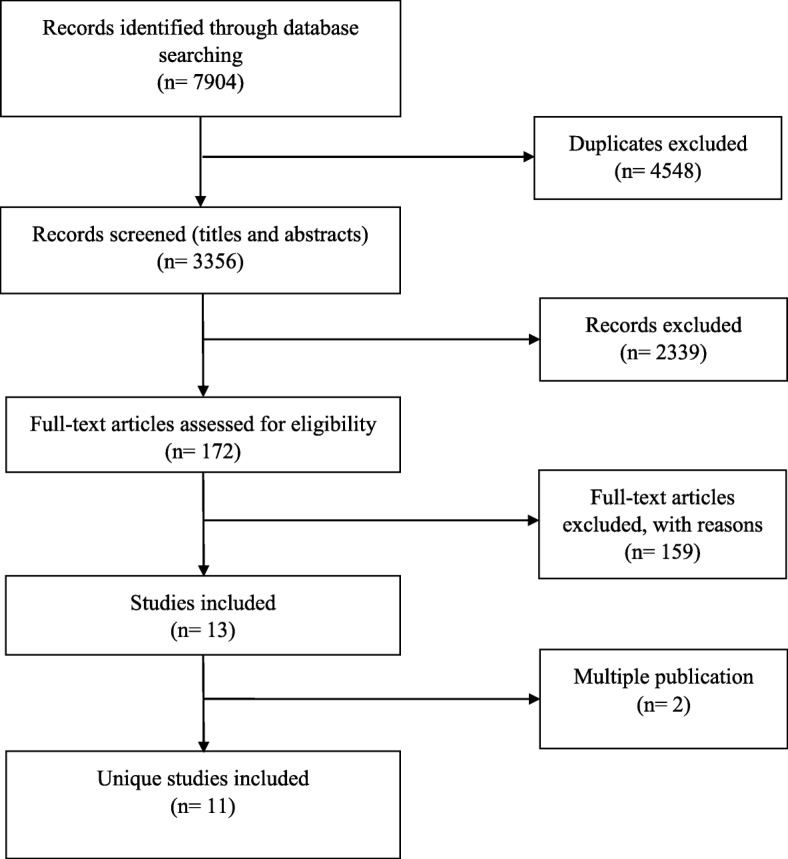
Preferred Reporting Items for Systematic Reviews and Meta-Analyses (PRISMA) flow diagram (From inception to Aug 10th, 2016)

A total of 7904 citations were identified by the search strategy, leaving 3356 citations after duplicate removal. After screening of titles and abstracts, 172 studies were assigned for full-text reading; of which only 13 met the eligibility criteria. Of these, 2 references were multiple publications; thus, analysis was conducted on 11 unique studies (six individual RCTs [46–51] and five cluster RCTs [52–56]) reported in 13 publications (see Fig. 1 for the Preferred Reporting Item for Systematic Reviews and Meta-Analyses (PRISMA) flow diagram). Additional files 3 and 4 report the characteristics of the included studies for professional and patient outcomes respectively.

The distribution of the included studies by country of origin was as follows: the Netherlands (*n* = 4) [49, 50, 52, 55], UK (*n* = 2) [48, 53], Australia (n = 2) [51, 54], USA (*n* = 1) [46], Switzerland (n = 1) [47], and Ireland (*n* = 1) [56]. Targeted MSK disorders included five studies on LBP [48, 52, 53, 55, 56], two studies on hip and knee osteoarthritis (OA) [49, 50], one on whiplash [54], two on neck pain [46, 51], and one on spinal disorders [47]. Two of the included studies tested KT interventions delivered in primary care settings [52, 53], two in private clinics [46, 54], two in both primary and secondary settings [49, 50], one in a conference for chiropractors [47]; one in primary care and occupational settings [48], one in communities of practice of physical therapists [55], and one in hospital outpatient physiotherapy clinics [56]. One study did not report their setting [51].

### Risk of bias

**Fig. 2:**
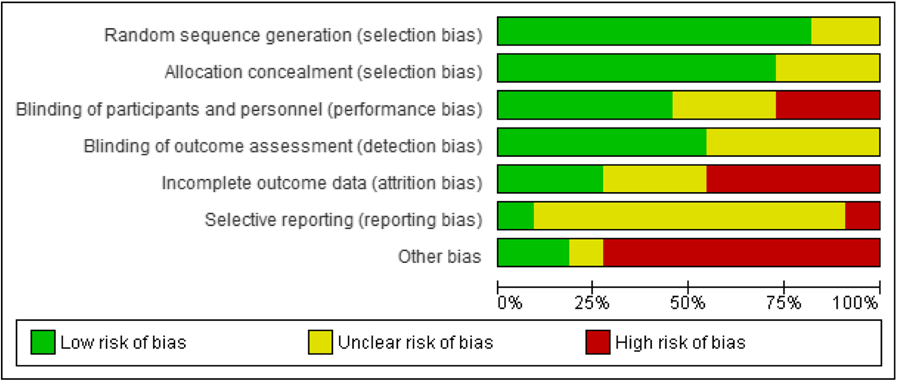
Risk of bias graph: review authors’ judgements about each risk of bias item presented as percentages across all included studies

**Fig. 3:**
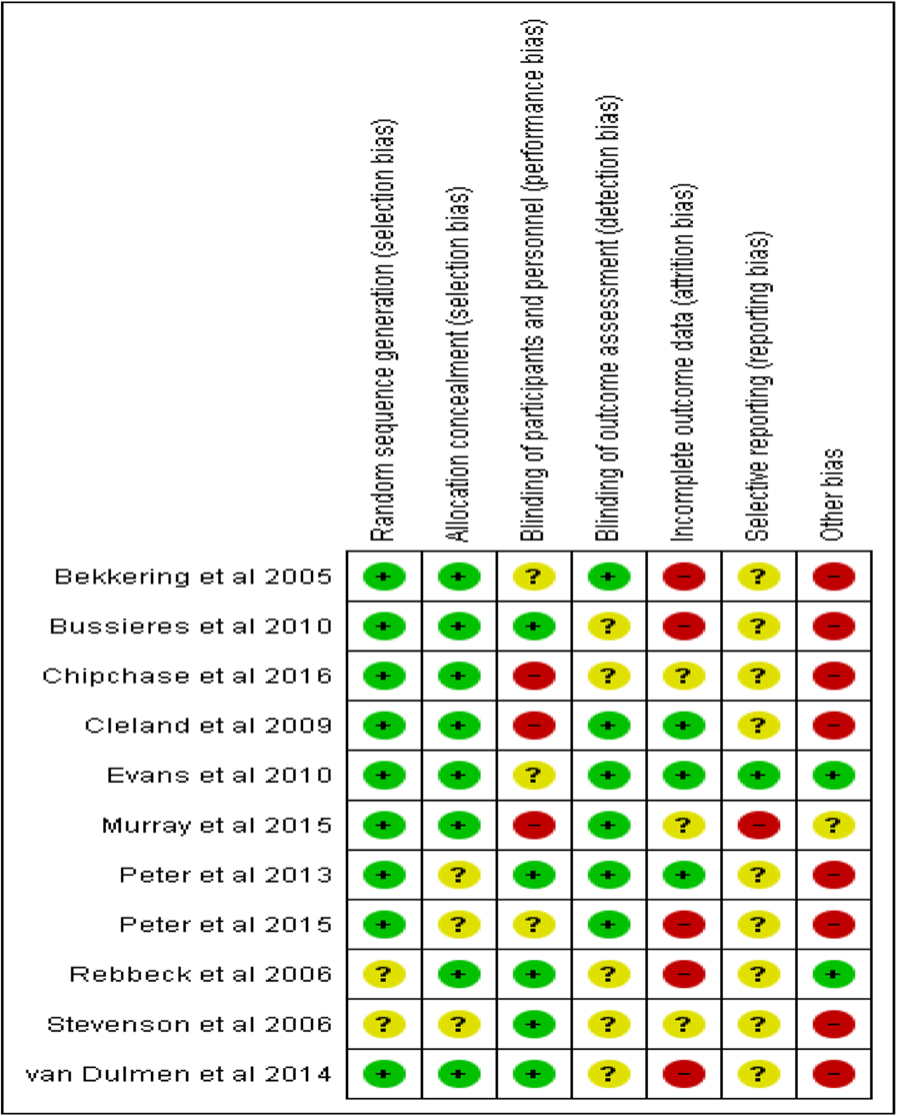
Risk of bias summary: review authors’ judgements about each risk of bias item for each included study

Figure 2 provides a graphical representation of the reviewers’ judgments on each ‘risk of bias’ item, which is presented as overall percentages across all included studies to facilitate comparison. Fig. 3 presents the reviewers’ judgments on all ‘risk of bias’ items for each included study. Over 75% of the included studies addressed random sequence generation (selection bias) and allocation concealment (selection bias); almost half of them carried out blinding of participants and personnel (performance bias) and blinding of outcome assessment (detection bias). One study was deemed to have a low risk of bias [46], six a moderate risk of bias [47–49, 52, 54–56], and four a high risk of bias [50, 51, 53]. Table 2 outlines the assessment of methodological quality (low, moderate, or high) of the included studies based on the EPOC standard criteria for reviews for RCTs.

**Table 2:** Methodological quality of controlled trials according to the EPOC method

Author (year)	Allocation concealment	Blinded assessment	Follow-up	Baseline measure	Reliable primary outcome	Protection against contamination	Summary^a^
Cleland (2009)	DONE	DONE	DONE	DONE	DONE	NC	Low
Evans (2010)	DONE	NC	DONE	DONE	NC	DONE	Moderate
Bussières (2010)	DONE	DONE	ND	DONE	NC	ND	Moderate
van Dulmen (2014)	DONE	NC	DONE	ND	ND	DONE	Moderate
Murray (2015)	DONE	DONE	NC	DONE	ND	DONE	Moderate
Peter (2013)	NC	DONE	DONE	DONE	NC	DONE	Moderate
Rebbeck (2006)	DONE	NC	NC	DONE	ND	DONE	Moderate
Peter (2015)	NC	NC	ND	NC	NC	DONE	High
Stevenson (2006)	NC	NC	NC	NC	NC	DONE	High
Bekkering (2005)	DONE	NC	NC	DONE	DONE	DONE	High
Chipchase (2016)	DONE	NC	NC	DONE	NC	NC	High

^a^Low risk of bias if the first 3 criteria are judged to be DONE; moderate if 1 or 2 of the first 3 criteria are judged to be NOT CLEAR (NC) or NOT DONE (ND); and high if more than 2 of the first 3 criteria are judged to be NOT CLEAR or NOT DONE

### Types of professional and patient outcomes

Ten studies [47–56] assessed MSK professional-related primary outcomes, including: knowledge about the guidelines [54, 55], self-confidence [51], adherence to guidelines [49, 50, 52], change in clinical practice (advice to patients [48, 53], and appropriate use of the diagnostic imaging [47]), and clinician-patient communication [56]. Each outcome was measured using a different scale. Four studies assessed patient-related outcomes, including patients’ function (disability) [46, 51, 52, 54] and pain [46, 52].

### Impact of KT interventions on professional and patient outcomes

Additional files 3 and 4 also report on the effectiveness of KT interventions on professional and patient outcomes respectively. Results were reported using effect sizes (Cohen’s *d*) and the reported *P*- values based on the original authors’ measurement units because there were no comparable outcome measures. Graphical representation of publication bias using a funnel plot was also not possible for this same reason.

#### Impact of KT interventions on professional outcomes

All studies included in this review involved KT interventions targeting professional outcomes, except one that focused on patient outcomes only [46]. Additional file 5 presents the elements of the KT interventions and use of theoretical frameworks, models or theories, while Table 3 presents the KT intervention types and their components.

**Table 3:** KT intervention types and their components

Intervention type		Control group	Number of comparison	Studies
	*Single component interventions vs. no intervention*			
Interactive educational meetings		No intervention	1	(Peter et al. 2015)
Distribution of educational materials		No intervention	1	(Evans et al. 2010)
	*Single component interventions vs. another intervention*			
Interactive educational meetings		Conventional educational meeting	1	(Peter et al. 2013)
	*Multi-component interventions vs. no intervention*			
Educational meetings + local opinion leaders		No intervention	1	(Stevenson et al. 2006)
Educational meetings + reminders		No intervention	2	(Bussières et al. 2010; Murray et al. 2015)
Educational meetings + Educational outreach visit		No intervention	1	(Cleland et al. 2009)
	*Multi-component interventions vs. another intervention*			
Interactive educational meeting+ Educational outreach visits + Distribution of educational materials + Local opinion leaders		Distribution of educational materials	1	(Rebbeck et al. 2006)
Interactive educational meetings + Distribution of educational materials		Distribution of educational materials	1	(Bekkering et al. 2005)
Educational meeting + Reminder		Educational meeting	1	(Chipchase et al. 2016)
Educational meeting + distribution of educational materials + other (peer-assessment)		Educational meeting + distribution of educational materials	1	(van Dulmen et al. 2014)

### Effects of single and multifaceted interventions on professional outcomes

#### Single-component KT interventions

In total three studies assessed the impact of single-component KT interventions on professional outcomes. Two of these tested a single-component KT intervention against no intervention [48, 50], while only one study compared a single-component KT intervention against another KT intervention [49]. Interactive educational meetings were found to have a small effect on enhancing professional adherence to clinical practice guidelines for hip and knee OA for physiotherapists, as compared to those who received no intervention (*d* = 0.23 (0.01–0.45), *P* < 0.05) [50] or a conventional educational meeting (*d* = 0.28 (0.02–0.53), *P* < 0.05) [49] at 3-month follow-up. Distribution of educational materials (LBP management guideline by postal mail) against no intervention also had a small effect on changing practice behavior (advice about physical activity level: *d* = 0.14 (0.05–0.23), *P* < 0.05; work: *d* = 0.16 (0.07–0.25), *P* < 0.05) for physiotherapists, chiropractors, and osteopaths at six months [48].

#### Multifaceted KT interventions

In total seven studies assessed the effectiveness of multifaceted KT interventions on professional outcomes. Three of these assessed multifaceted KT interventions were against no intervention [47, 53, 56] and reported mixed results. The first study showed that communication skills training from educational meetings and reminders for physiotherapists to support patients’ psychological needs were found to be more effective than the control for managing chronic LBP at 16.7 ± 6.9 weeks (*d* = 2.27 (1.24–3.29), *P* < 0.05) [56]. A second study reported on the effect of educational meetings on enhancing the appropriate use of diagnostic imaging for spinal disorders for chiropractors [47]. While the subgroup with access to a reminder at midpoint performed significantly better than another subgroup (F = 4.486; *df* = 1, 30; *P* = 0.043), the overall scores for the pretest and the final test did not significantly differ at 10 weeks (d = 0.05 (− 0.26–0.36), *P* > 0.05). A third study combined interactive educational meetings with local opinion leaders against no intervention and was also found to be ineffective in changing physiotherapists’ clinical practice regarding the use of best evidence on ‘Psychosocial Yellow Flags’ for non-specific LBP patients at 6 months [53] (see Additional file 3 for the summary effect sizes).

The other four studies compared multifaceted KT interventions against other single or multifaceted interventions [51, 52, 54, 55] and reported positive findings. A combination of interactive educational meetings, educational outreach visits, distribution of educational materials, and local opinion leaders showed a significantly superior effect (*d* = 2.151 (1.2–3.1), *P* < 0.05) compared with the distribution of educational materials (guideline by postal mail), in changing physiotherapists’ knowledge about the whiplash guideline at 12 months [54]. Implementation of the Dutch physical therapy guideline for LBP found that educational meetings, distribution of educational materials, and peer-assessment compared to educational meetings and distribution of educational materials significantly improved knowledge and guideline-consistent clinical reasoning with a large effect (*d* = 0.824 (− 0.23–1.88), *P* < 0.05) at 6 months [55]. Interactive educational meetings and distribution of educational materials also had a significant, but small effect (*d* = 0.4 (0.08–0.71), *P* < 0.05) compared to distribution of educational materials (guideline by postal mail) in improving physiotherapists’ adherence to a non-specific LBP guideline at 1 month [52]. One other study found that a traditional continuing professional development educational meeting and a reminder compared to a traditional educational meeting alone had a small, non-significant effect in improving physiotherapists’ practice behaviour or confidence in using multimodal interventions (advice, education, exercise and manual therapy) for neck pain patients at 2 months [51].

Together, these findings suggest that for professional outcomes, single-component KT interventions are more effective than no intervention, and multifaceted interventions are more effective than single-component interventions.

### Impact of KT interventions on patient outcomes

Four studies evaluated the impact of the KT interventions on patient outcomes [46, 51, 52, 54].

#### Single-component KT interventions

No study assessed the impact of a single-component KT intervention on patient outcomes.

#### Multifaceted KT interventions

Four studies assessed the effectiveness of multifaceted KT interventions on patient outcomes. One study favoured a multifaceted intervention (combination of educational meetings and an educational outreach visit) over no intervention for reducing neck disability at 12 months (*d* = 0.27 (0.09–0.45), *P* < 0.05), but not level of neck pain (*d* = 0.16 (− 0.01–0.33), *P* > 0.05) [46]. Three other studies investigated the effectiveness of multifaceted interventions against other types of interventions. Interactive educational meetings were predominantly shared with all of these studies along with other components. A combination of distribution of educational materials about a non-specific LBP guideline and interactive educational meetings against distribution of educational materials alone had no effect on improving disability (*d* = 0.28 (0.10–0.45), *P* > 0.05) or pain at 6, 12, 26, and 52 weeks (*d* = 0.31 (0.13–0.49), *P* > 0.05) [52]. A combination of interactive educational meetings, educational outreach visits, local opinion leaders, and distribution of educational materials (a guideline on the management of acute whiplash) was not superior to the distribution of educational materials alone for improving disability at 12 months (*d* = 0.06 (− 0.39–0.51), *P* > 0.05) [54]. Lastly, a combination of educational meetings and reminders was ineffective for improving disability for neck pain patients at 4 months (*d* = 0.11 (− 0.25–0.48), *P* > 0.05) [51].

Overall, included studies suggest that multifaceted interventions delivered to professionals did not improve patient outcomes.

## Discussion

This review summarized the evidence from eleven studies that investigated the impact of various KT interventions on MSK professional and patient outcomes for MSK disorders. Nine studies involved physiotherapists, one chiropractors, and one a mixed of physiotherapists, chiropractors, and osteopaths. The targeted behaviors were the general management of MSK disorders (nine studies), diagnostic spine imaging, and professional-patient communication. Five studies were on LBP, two on neck pain and whiplash, one on spinal disorders, and two others on OA of the hip and knee.

Although this review included only RCTs, the majority of the included studies were considered to have moderate-to-high risk of bias. This is consistent with the findings of similar reviews [19, 29, 57, 58]. The assessment of the risk of bias was challenging due to poor or incomplete reporting of methodological characteristics in several studies. The small number of eligible studies prevented us from comparing outcome measures across studies considering this review included multiple KT strategies, professions, targeted behaviours and MSK conditions.

Educational meetings were used across most the included studies. Three studies suggested that single-component interventions had a small, albeit significant effect for improving professional outcomes whether compared to no intervention [48, 50] or another intervention [50].

The majority of the included studies (8/11) used multifaceted KT interventions. This is consistent with other reviews in similar areas [19, 58], possibly because the use of multifaceted interventions was previously encouraged [59]. Seven studies assessed the effectiveness of multifaceted interventions on professional outcomes. Three of these were compared to no interventions showing mixed effects for educational meetings and reminders [47, 56], and no effect for interactive educational meetings delivered by local opinion leaders [53]. Four other studies were compared to single-type interventions, with three showing favorable results [52, 54, 55], and one a non-significant trend toward improvement t [51]. While our findings are consistent with seven prior reviews [19, 29, 30, 57, 58, 60, 61] suggesting multifaceted interventions are more effective than single-component interventions, a recent overview of systematic reviews found that multifaceted interventions are no more effective than single-component interventions [62]. However their findings should be interpreted with caution considering the following limitations: First, the authors limited their search to reviews available on the Rx-for-Change database. Second, they did not search the ‘grey literature’, possibly omitting other relevant work in the field. Third, they did not retrieve data from the original studies that comprised the included reviews, thereby having to rely on the information reported by the review authors. Fourth, they relied on reviews that mainly used non-statistical analyses, so they did not account for the effect sizes of individual studies. Fifth, the included reviews were comprised of different methodological designs (i.e. RCTs, controlled trials, interrupted time series, etc.), whereas this review considered only RCTs.

Finally, four included studies suggested that multifaceted interventions delivered to professionals were ineffective in improving patient outcomes [46, 51, 52, 54]. Similar findings were reported by other reviews targeting physiotherapists [30, 32]. Bekkering et al. (2005) [52] and Rebbeck et al. (2006) [54] attributed the lack of effect of KT interventions on patient outcomes to the high quality care delivered by physiotherapists mitigating further improvement in patient health. Other authors suggested instead this may be due to unmeasured patient’s characteristics (e.g., fear avoidance and depression) moderating the effect on patient outcomes [46], or to the small effect of individual components include in the KT interventions evaluate in these RCTs [51]. For instance adding outreach visits may increase the likelihood of improving outcomes.

About half of the included studies indicated using theoretical frameworks, theories or models to guide the design of the behavior change intervention [49, 50, 52, 53, 56]. Only one of the studies provided a rationale for choosing a specific theory and a description of how using such theories informed intervention design [56]. This is in part because KT frameworks had not been developed when some of the primary RCTs were designed.

Findings from this review suggest that multifaceted educational interventions appear to be effective for improving professional outcomes. However, several elements ought to be improved in future trials to increase our confidence regarding the effect of multifaceted interventions, including: better reporting of providers’ characteristics and level of training; theoretical framework used to support behavior change; intervention components; and the implementation fidelity. Larger sample sizes and a clear rationale for selecting each component of the KT intervention are also needed [33].

Because of the nature and the scope of practice of each MSK profession, the success of specific KT interventions for one profession may not necessarily be successfully replicated among other health professions [19]. Improving the reporting of intervention elements may help explain why certain KT interventions are effective or ineffective, in particular with respect to multifaceted components, and whether more effective interventions are likely to also work with other MSK professionals or for other disorders [59]. Mapping behavior change techniques to previously identified barriers when designing KT interventions may improve the likelihood of successful professional behaviour change and improve patient health outcomes [63–66].

The strength of this review stemmed from the rigorous search strategy it employed. It captured more studies than antecedent reviews [19, 29, 57, 58] within relevant areas over similar periods. The search strategy we used was recently validated and recommended by Cochrane and Rx for change, helping capture studies other reviews did not identify [48, 49]. Moreover, the present study covered a wide spectrum of MSK disorders and health care professionals. To our knowledge, this is the first review to report on the use of single-component interventions from RCTs for MSK disorders. Other reviews reported on the use of single-component interventions in non-RCT designs [19, 29, 57, 58]. Furthermore, other reviews had not reported the intervention details, for instance, Ospina et al. (2013) [57] classified both Bekkering et al. (2005) [52] and Stevenson et al. (2006) [53] under single-component strategies when in fact, these should be classified under multifaceted strategies as also reported in other reviews [19, 29, 58]. The authors may have overestimated the effect of multifaceted interventions in those reviews.

### Study limitations

Despite these strengths, this review has several limitations. First, the search was restricted to studies published in English. Second, studies were mainly conducted in western countries which may restrict the generalizability of our findings. Third, a graphical representation of the publication bias using funnel plots was not possible as there were no comparable outcome measures. However, this review controlled for the effect of multiple publication biases in terms of the results. This is important as studies with significant findings often have multiple publications [67] which could lead to overestimation of the intervention’s impacts in reviews [68, 69]. We identified two multiple publications we presented as a single study. Data extraction was not double-blinded which may be a source of bias. This review did not identify any pragmatic studies. Fourth, our review included RCTs only to minimize bias and confounding. Nonetheless, RCTs are not without limitations (e.g., population availability, contamination, time for follow-up, external validity, cost) [70]. The integration of multiple study designs could have better informed ‘real-world’ clinical practice and the many facets of patient relevant issues [71]. Fifth, patient reported outcomes concepts/domains and time points for assessment should closely align with the trial objectives and hypotheses [72]. The use of measures such as health-related quality of life and coping may be more relevant and sensitive indicators of success in KT trials than specific symptoms such as pain.

## Conclusion

This review is one of the first to summarize studies on the effectiveness of KT interventions to improve the uptake and application of clinical practice guidelines and best practices specific to MSK disorders among a wide range of MSK professionals. The findings showed that primary trials use a variety of educational approaches with favorable but inconsistent impact on professional outcomes. Very few studies reported the effect on patient outcomes and results tended to be negative. The overall quality of included RCTs was poor. There was poor reporting of the interventions and a lack of reporting the way theoretical frameworks were implemented in the included trials.

## Additional files

Additional file 1: Search strategy. (PDF 264 kb)

Additional file 2: Characteristics of excluded studies. (PDF 463 kb)

Additional file 3: The main characteristics of KT interventions and their effectiveness of the professional outcomes. (DOCX 29 kb)

Additional file 4: The main characteristics of KT interventions and their effectiveness on patient outcomes. (DOCX 18 kb)

Additional file 5: Elements of KT interventions and use of theoretical frameworks, models or theories. (DOCX 31 kb)

## Abbreviations

KT: Knowledge translation
LBP: Low back pain
MSK: Musculoskeletal
OA: Osteoarthritis
RCT: Randomized controlled trials
