# Supplementary material for: The effectiveness of interventions designed to increase the uptake of clinical practice guidelines and best practices among musculoskeletal professionals: a systematic review
Source: BMC Health Serv Res. 2018 Jun 8;18:435. doi: 10.1186/s12913-018-3253-0 (PMC5994025; doi:10.1186/s12913-018-3253-0)
Supplement: Supplementary file 1 — Search strategy. (PDF 264 kb) [file 12913_2018_3253_MOESM1_ESM.pdf]

Appendix 1. Search strategy

MEDLINE search strategy

1. Physical Therapy Modalities/
2. "Physical Therapy (Specialty)"/
3. (physical therap* or physiotherap*).mp.
4. occupational therapy/ or occupational therap*.mp.
5. Manipulation, Chiropractic/ or Chiropractic/
6. chiropract*.tw.
7. chiroprax*.tw.
8. chiropractic.mp.
9. exp Osteopathic Medicine/
10. OMT.mp.
11. Osteopathic.mp. or Manipulation, Osteopathic/ or Osteopathic Physicians/ or Osteopathic Medicine/
12. Osteopath$.ti,ab.
13. (Osteopath$ adj3 manipulat$).mp.
14. manual therap*.mp.
15. sport* therap*.mp.
16. sport physician*.mp.
17. Sports medicine/
18. Exercise Therap*/ or Exercise physiolog*.mp.
19. Massage therap*.mp.
20. kinesiolog*.mp.
21. Physiatr*.mp.
22. "Physical and Rehabilitation Medicine"/
23. Physical medicine.mp.
24. rehabilitation medicine.mp.
25. orthoped*.mp.
26. orthopaed*.mp.
27. orthopod*.mp.
28. Doctor of Podiatric Medicine.mp.
29. Podiatr*.mp.
30. Chiropod*.mp.
31. hand therap*.mp.
32. foot therap*.mp.
33. Acupunctur*.mp.
34. Acupuncture Therapy/ or Acupuncture/
35. Acupressure/
36. or/1-35
37. exp back pain/
38. neck pain/
39. Shoulder pain/
40. Tennis Elbow/
41. exp Tendinopathy/
42. Whiplash Injuries/
43. Sciatica/

44. Intervertebral Disk Displacement/
45. (pain adj3 (neck or back or shoulder? or elbow? or forearm? or wrist? or hand? or arm? or hip? or knee? or ankle? or leg? or foot or feet)).tw.
46. (epicondylitis or tendonitis or tendinitis or bursitis or synovitis or sprain? or strain?).tw.
47. exp Joint Diseases/
48. exp Spinal Diseases/
49. exp Spondylarthritis/
50. exp osteoarthritis/
51. exp Osteoporosis/
52. (arthriti$ or osteoarthriti$ or osteoporo$ or bone loss$).tw.
53. ankle/ or hip/ or knee/
54. elbow/ or wrist/ or shoulder/
55. elbow joint/ or exp hand joints/ or hip joint/ or knee joint/ or sacroiliac joint/ or shoulder joint/
56. exp cervical vertebrae/ or intervertebral disk/ or lumbar vertebrae/ or thoracic vertebrae/
57. exp Back/
58. exp Spine/
59. (spine or spinal).tw.
60. Musculoskeletal Disease*.mp.
61. or/37-60
62. (intervention? adj3 (clinic? or clinician? or collaborat$ or community or complex or education$ or effect$ or financial or impact? or improv$ or individuali?e? or individuali?ing or inpatient? or multifacet$ or multi-facet$ or organi?ation$ or outpatient? or personali?e? or personali?ing or allied healthcare or therapist? or physician? or practitioner? or professional$ or provider? or regulatory or structured or tailor$ or target$ or interdisciplin$ or multi-disciplin$ or multidisciplin$ or team$)).ti.
63. (intervention? adj3 (clinician? or collaborat$ or community or complex or doctor? or educational or financial or impact? or individuali?e? or individuali?ing or multifacet$ or multi-facet$ or organi?ation$ or personali?e? or personali?ing or physician? or allied healthcare or therapist? or practitioner? or professional$ or provider? or regulatory or structured or tailor$ or target$ or interdisciplin$ or multi-disciplin$ or multidisciplin$ or team$)).ab.
64. (integrating or integration).ti.
65. (Medical Order Entry Systems/ or medical records/ or health records, personal/ or medical record linkage/ or medical records, problem-oriented/ or medical records systems, computerized/ or electronic health records.mp.) and (intervention? or improv$ or effectiv$ or "patient care" or quality or implement$).ti.
66. ((computeri$ or doctor? or allied healthcare or therapist? or medical or physician? or practitioner? or provider) adj2 (order adj2 (system? or entry))).ti,ab.
67. (((evidence or evidence-based) adj4 intervention) or evidence-driven).ti,ab.
68. "practice-based".ti,ab.
69. "delivery of health care"/
70. ((effectiv$ or improv$) adj3 (adheren$ or care or clinician? or therapist? or decision$ or doctor? or implement$ or health care or healthcare or initiative? or inpatient? or management or multifacet$ or multi-component or out-patient? or patient-led or patient-based or physician? or practi?e? or practitioner? or preventiv$ or preventativ$ or professional? or program? or programme? or provider? or unit or units)).ti.

71. ((effectiv$ or improv$) adj2 (patient-care or decision$ or therapist? or doctor? or implement$ or health care or healthcare or initiative? or management or practitioner? or provider? or (primary care or primary medical care or primary health care or primary patient care))).ab.
72. recommended practice?.ti,ab.
73. ((information or evidence) adj2 uptake).ti,ab.
74. ((knowledge adj2 (application or broke$ or creation or diffus$ or disseminat$ or exchang$ or implement$ or management or mobili$ or translat$ or transfer$ or uptake or utili$)) or (evidence$ adj2 (exchang$ or translat$ or transfer$))).ti,ab.
75. (KT adj2 (application or broke$ or diffus$ or disseminat$ or decision$ or exchang$ or implement$ or intervent$ or mobili$ or plan$ or policy or policies or strateg$ or translat$ or transfer$ or uptake or utili$)).ti,ab.
76. ((utili?e? or utili?ing or implement$) adj2 (research or evidence)).ti,ab.
77. ((evidence or intervention?) adj3 effectiveness).ti,ab.
78. ((research or evidence) adj2 action).ti,ab.
79. ((evaluat$ or facilitiat$ or impact or incorporat$ or policy or practice or translat$) adj2 evidence).ti,ab.
80. (health policy or policy maker? or decision maker? or (policy adj3 evidence)).ti,ab.
81. ((computer-tailored or individuali?ing or individuali?ed or personali?e? or personali?ing or tailor$) adj2 (feedback or intervention? or information or plan?)).ti,ab.
82. ((evidence or identify or identified or practice) adj3 gap?).ti,ab.
83. (change adj2 (barrier? or faciliat$)).ti,ab.
84. (leaflet$ or pamphlet$ or "written information").ti,ab.
85. (academic detailing or e-detailing or (opinion? adj2 leader?)).ti,ab.
86. ("audit and feedback" or (feedback adj2 prompt$) or ((physician? or allied healthcare or therapist? or doctor? or practitioner? or provider?) adj feedback)).ti,ab.
87. reminder?.ti,ab. or Reminder systems/
88. ((doctor? or physician? or allied healthcare or therapist? or practitioner?) adj2 behavio?r?).ti,ab.
89. (practice pattern? or ((change? or changing) adj2 practice)).ti,ab.
90. Physician's Practice Patterns/ and (change? or changing or improv$ or impact or effect$ or reduce? or reducing or increase? or increasing or otimi?e? or optimal or quality or healthcare or patient care or chronic or management or administration).ti,hw.
91. (primary care or primary medical care or primary health care or primary patient care).ti.
92. ((doctor? or allied healthcare or therapist? or physician?) adj2 role?).ab.
93. *"Referral and Consultation"/
94. (Referral? and (primary care or primary medical care or primary health care or primary patient care or specialist? or practitioner? or change? or changing or improv$ or impact or effect$ or reduce? or reducing or increase? or increasing or optimi?e? or optimal or quality or healthcare or therapist? or patient care or intensive care or chronic or management or administration)).ti,hw.
95. ((dual or enhanced or extend$) adj2 role?).ti,ab. and (health personnel/ or allied health personnel/ or exp therapist/ or exp physicians/)
96. ((dual or enhanced or extend$) adj2 role?).ti,ab. and (physician? or practitioner? or resident? or intern? or primary care or primary medical care or primary health care or primary patient care or allied healthcare or therapist?).ti,hw.
97. ((physician? or allied healthcare or therapist? or provider?) adj2 initiat$).ti,ab.

98. exp education, continuing/ and (improv$ or impact or care or effective therapy or healthcare or quality).ti,hw.
99. (((continuing or "on the job" or "off the job" or postgrad$ or post-grad$ or resident? or intern? or internship? or workplace) adj2 (education$ or training)) or (skill? adj (education or training))).ti,ab.
100. (virtual reality or VR Training or VR simulat$ or (simulat$ adj2 skill?)).ti,ab.
101. simulat$.ti. and (education$.ti,fs,hw. or teaching.ti,hw. or learning.ti,hw.)
102. (blog$ or wiki$ or PDA or "palm pilot?" or blackberr$ or Twitter or tweet or tweeting or facebook or social networking or social marketing or youtube).ti,ab. or blogging/
103. (health 20 or healthcare 20 or health care 20 or web 20).ti,ab.
104. *guideline adherence/ or (guideline? adj2 (adherence or implement$ or concordance)).ti,ab.
105. (self-monitor$ or self-care or self-manag$ or (home-based or home-managed)).ti,ab.
106. (lay health$ worker? or community health$ worker?).ti,ab.
107. (community adj2 (care or health or healthcare or therapist? or project? or program$ or practice? or physician? or doctor?)).ab. or "community-based".ti.
108. (((individuali$ or integrated) adj2 (care or healthcare or medical care)) or patient-centred or patient-centered or patient-control$).ti,ab.
109. ((performance$ adj2 pay$) or (P4P or "pay for quality improvement?" or P4QI or "fee-for service?" or "payment? by result?" or capitated or capitation) or ((payment? or fee or fees) adj2 (mix or mixed))).ti,ab.
110. quality improvement.ti,ab.
111. ((barrier? or improve? or improving or increase? or increasing or insurance or insurer? or intervention? or optim$ or promot$ or sub-optimal$) adj4 (patient adherence or patient compliance)).ab. or ((barrier? or improve? or improving or increase? or increasing or insurance or insurer? or intervention? or optim$ or promot$ or sub-optimal$) and (patient adherence or patient compliance)).ti.
112. ((barrier? or improv$ or increas$ or promot$) adj3 (adopt$ or implement$)).ti,ab.
113. (*Telemedicine/ or (e-health$ or e-medicine or e-practice or e-practition$ or Tele-diagnos$ or tele-consult$ or tele-health$ or tele-medicine or telemedicine or telehealth$ or telemedicine or tele-medicine or telemonitoring or tele-monitoring or telerehab$ or tele-rehab$).ti,ab.) and (impact? or improv$ or change? or changing or effect$ or quality).ti,hw.
114. Patient compliance/ and (improv$ or impact or care or effective or healthcare or quality).ti,hw.
115. ((patient or treatment) adj (adherence or nonadherence or non-adherence or compliance or non-compliance)).ab. or ((patient or treatment) adj2 (adherence or nonadherence or non-adherence or compliance)).ti.
116. or/62-115
117. Randomized Controlled Trials as Topic/
118. randomized controlled trial/
119. Random Allocation/
120. Double Blind Method/
121. Single Blind Method/
122. clinical trial/
123. clinical trial, phase iii.pt.
124. clinical trial, phase iv.pt.
125. controlled clinical trial.pt.

126. randomized controlled trial.pt.
127. multicenter study.pt.
128. clinical trial.pt.
129. exp Clinical Trials as topic/
130. or/117-129
131. (clinical adj trial$).tw.
132. ((singl$ or doubl$ or treb$ or tripl$) adj (blind$3 or mask$3)).tw.
133. PLACEBOS/
134. placebo$.tw.
135. Randomly allocated.tw.
136. (allocated adj2 random$).tw.
137. or/131-136
138. 130 or 137
139. case report.tw.
140. letter/
141. historical article/
142. or/139-141
143. 138 not 142
144. 36 and 61 and 116 and 143

EMBASE search strategy
1. (primary care or primary medical care or primary health care or practitioner? or allied healthcare or therapist? or primary patient care).ti.
2. (intervention? adj3 (clinic? or clinician? or collaborat$ or community or complex or education$ or effect$ or financial or impact? or improv$ or individuali?e? or individuali?ing or inpatient? or multifacet$ or multi-facet$ or allied healthcare or therapist? or interdisciplin$ or multi-disciplin$ or multidisciplin$ or organi?ation$ or outpatient? or personali?e? or personali?ing or physician? or practitioner? or professional$ or provider? or regulatory or structured or tailor$ or target$ or team$)).ti.
3. (intervention? adj3 (clinician? or collaborat$ or community or complex or doctor? or educational or financial or impact? or individuali?e? or individuali?ing or multifacet$ or multi-facet$ or allied healthcare or therapist? or interdisciplin$ or multi-disciplin$ or multidisciplin$ or organi?ation$ or personali?e? or personali?ing or physician? or practitioner? or professional$ or provider? or regulatory or structured or tailor$ or target$ or team$)).ab.
4. (integrating or integration).ti.
5. (medical record/ or electronic medical record/) and (intervention? or improv$ or effectiv$ or "patient care" or quality or implement$).ti.
6. order entry.ti. or (order entry adj2 (physician? or doctor? or provider? or computer$ or system? or process$)).ab.
7. ((computeri$ or doctor? or medical or allied healthcare or therapist? or physician? or practitioner? or provider or prescrib$) adj2 (order adj2 (system? or entry))).ti,ab.
8. (((evidence or evidence-based) adj4 intervention) or evidence-driven).ti,ab.
9. "practice-based".ti,ab.
10. *health care delivery/ or (delivery of health care or health care delivery).ti.
11. ((CHANGE? or changing or improv$ or effect$ or influenc$) and (care or health$ or practice)).ti.

12. ((care adj2 (continuity or integrated or integrative or shared)) and (chang$ or collaborat$ or community$ or cooperat$ or co-operat$ or effect? or impact? or improv$ or implement$ or team$ or transform$)).ti.
13. (care adj2 (continuity or integrated or integrative or shared) adj4 (chang$ or collaborat$ or community$ or cooperat$ or co-operat$ or effect? or impact? or improv$ or implement$ or team$ or transform$)).ti.
14. restructuring.ti. or (restructur$ adj4 (care or health or patient? or physician? or organi?ational or allied healthcare or therapist? or doctor?)).ti,ab.
15. ((effectiv$ or improv$) adj3 (adheren$ or care or clinician? or decision$ or doctor? or implement$ or health care or healthcare or initiative? or inpatient? or management or therapist? or interdisciplin$ or multi-disciplin$ or multidisciplin$ or multifacet$ or multi-facet$ or multi-component or out-patient? or patient-led or patient-based or physician? or practi?e? or practitioner? or prescrib$ or prescription? or preventiv$ or preventativ$ or professional? or program? or programme? or provider? or unit or units)).ti.
16. ((effectiv$ or improv$) adj2 (patient-care or decision$ or doctor? or implement$ or health care or healthcare or therapist? or interdisciplin$ or multi-disciplin$ or multidisciplin$ or initiative? or management or practitioner? or provider? or (primary care or primary medical care or primary health care or primary patient care))).ab.
17. recommended practice?.ti,ab.
18. ((information or evidence) adj2 uptake).ti,ab.
19. ((knowledge adj2 (application or broke$ or creation or diffus$ or disseminat$ or exchang$ or implement$ or management or mobili$ or NETWORK? or translat$ or transfer$ or uptake or utili$)) or (evidence$ adj2 (exchang$ or translat$ or transfer$))).ti,ab.
20. (KT adj2 (application or broke$ or diffus$ or disseminat$ or decision$ or exchang$ or implement$ or intervent$ or mobili$ or NETWORK? or plan$ or policy or policies or strateg$ or translat$ or transfer$ or uptake or utili$)).ti,ab.
21. ((utili?e? or utili?ing or implement$) adj2 (research or evidence)).ti,ab.
22. ((evidence or intervention?) adj3 effectiveness).ti,ab.
23. ((research or evidence) adj2 action).ti,ab.
24. ((evaluat$ or facilitiat$ or impact or incorporat$ or policy or practice or translat$) adj2 evidence).ti,ab.
25. (health policy or policy maker? or decision maker? or (policy adj3 evidence)).ti,ab.
26. ((computer-tailored or individuali?ing or individuali?ed or personali?e? or personali?ing or tailor$) adj2 (feedback or intervention? or information or plan?)).ti,ab.
27. ((evidence or identify or identified or practice) adj3 gap?).ti,ab.
28. (change adj2 (barrier? or faciliat$)).ti,ab.
29. (collaborative? or skill-mix).ti.
30. (collaborative? or interdisciplin$ or inter-disciplin$ or INTRA-DISCIPLINARY or INTRADISCIPLINARY or multidisciplin$ or multi-disciplin$ or team? or team-based or skill-mix).ti.
31. (((interdisciplinary or intradisciplinary or intra-disciplinary or collaborative) adj research) or ((collaborative or intradisciplinary or intra-disciplinary or multidisciplinary or interdisciplinary) adj2 (care or team? or unit))).ab.
32. action plan?.ti.
33. (BROCHURE? or pamphlet$ or (written adj2 (information or material?))).ti,ab.

34. (LEAFLET? and (care or consumer? or allied healthcare or therapist? or doctor? or education$ or information$ or label$ or packag$ or physician? or practitioner? or provider? or Rx)).ti.
35. (LEAFLET? adj3 (care or consumer? or doctor? or allied healthcare or therapist? or education$ or evidence-based or information$ or label$ or packag$ or physician? or practitioner? or provider? or Rx)).ab.
36. (academic detailing or e-detailing or (opinion? adj2 leader?)).ti,ab.
37. ("audit and feedback" or (feedback adj2 prompt$) or ((physician? or doctor? or allied healthcare or therapist? or practitioner? or nurse? or provider?) adj feedback)).ti,ab.
38. reminder?.ti. or (reminder? adj3 (system? or paper$ or computer$ or physician? or allied healthcare or therapist? or primary care or professional? or practitioner?)).ab. or reminder system/
39. ((CLINICIAN? or doctor? or physician? or allied healthcare or therapist? or practitioner?) adj2 behavio?r?).ti,ab.
40. (Practice Pattern? or PHYSICIAN? PRACTICE or allied healthcare practice or therapist? practice).ti,ab.
41. ((doctor? or allied healthcare or therapist? or physician?) adj2 role?).ab.
42. (Referral? adj2 (PHYSICIAN? or therapist? or primary care or primary medical care or primary health care or primary patient care or change? or changing or improv$ or impact or effect$ or reduce? or reducing or increase? or increasing or optimi?e? or optimal or quality or healthcare or patient care or chronic or management or administration)).ab.
43. (Referral? and (PHYSICIAN? or therapist? or primary care or primary medical care or primary health care or primary patient care or change? or changing or improv$ or impact or effect$ or reduce? or reducing or increase? or increasing or optimi?e? or optimal or quality or healthcare or patient care or chronic or management or administration)).ti.
44. (CONSULTATION? or COLLABORATION).ti.
45. DECISION support system/
46. DECISION SUPPORT.ti,ab.
47. ((dual or enhanced or extend$) adj2 role?).ti,ab. and (exp allied healthcare/ or exp therapist/ or exp physician/)
48. (((dual or enhanced or extend$) adj role?) and (doctor? or allied healthcare or therapist? or physician? or practitioner? or resident? or intern? or primary care or primary medical care or primary health care or primary patient care or practitioner?)).ti.
49. ((DUAL or enhanced or extend$) adj role? adj4 (doctor? or allied healthcare or therapist? or physician? or practitioner? or resident? or intern? or primary care or primary medical care or primary health care or primary patient care or practitioner?)).ab.
50. ((physician? or allied healthcare or therapist? or provider?) adj2 initiat$).ti,ab.
51. EDUCATION, CONTINUING/ and (improv$ or impact or care or effective or healthcare or quality or primary care or primary medical care or primary health care or primary patient care).ti.
52. (((continuing or "on the job" or "off the job" or postgrad$ or post-grad$ or resident? or intern? or internship? or workplace) adj2 (education$ or training)) or (skill? adj (education or training))).ti,ab.
53. (virtual reality or VR Training or VR simulat$ or (simulat$ adj2 skill?)).ti,ab.
54. (simulat$ and (EDUCATION$ or teaching or learning)).ti.
55. (blog$ or wiki$ or PDA or "palm pilot?" or blackberr$ or Twitter or tweet or tweeting or facebook or social networking or social marketing or youtube).ti,ab.

56. (health 20 or healthcare 20 or health care 20 or web 20).ti,ab.
57. (guideline? adj2 (adherence or implement$ or concordance)).ti,ab.
58. (self-monitor$ or self-care or self-manag$ or (home-based or home-managed)).ti,ab.
59. (lay health$ worker? or community health$ worker?).ti,ab.
60. (community and (care or health or healthcare or therapist? or program$ or practice? or physician? or project? or doctor?)).ti. or (community adj2 (care or health or healthcare or project? or program$ or physician? or therapist? or doctor?)).ab. or "community-based".ti.
61. (((individuali$ or integrated) adj2 (care or healthcare or medical care)) or patient-centred or patient-centered or patient-control$).ti,ab.
62. (((FINANCIAL or salary or payment? or fee-structure?) adj2 incentive?) or reference pricing).ti,ab.
63. ((performance$ adj2 pay$) or target payment? or (P4P or "pay for quality improvement?" or P4QI or "fee-for service?" or fee-based or "payment? by result?" or capitated or capitation) or ((co-pay$ or payment? or fee or fees) adj2 (cap or capped or mix or mixed))).ti,ab.
64. quality improvement.ti,ab.
65. ((barrier? or improve? or improving or increase? or increasing or insurance or insurer? or intervention? or optim$ or promot$ or sub-optimal$) adj4 (patient adherence or patient compliance)).ab. or ((barrier? or improve? or improving or increase? or increasing or insurance or insurer? or intervention? or optim$ or promot$ or sub-optimal$) and (patient adherence or patient compliance)).ti.
66. ((barrier? or improv$ or increas$ or promot$) adj3 (adopt$ or implement$)).ti,ab.
67. (MOBILE adj2 (health or care)).ti,ab.
68. exp *Telemedicine/ or (E-HEALTH$ or telemanag$ or tele-manag$ or e-medicine or e-practice or e-practition$ or Tele-diagnos$ or tele-consult$ or tele-health$ or tele-medicine or telemedicine or telehealth$ or telemedicine or tele-medicine or telemonitoring or tele-monitoring or telerehab$ or tele-rehab$).ti.
69. (E-HEALTH$ or e-medicine or e-practice or e-practition$ or Tele-diagnos$ or tele-consult$ or tele-health$ or telemanag$ or tele-manag$ or tele-medicine or telemedicine or telehealth$ or telemedicine or tele-medicine or telemonitoring or tele-monitoring or telerehab$ or tele-rehab$).ti,ab. and (impact? or improv$ or change? or changing or effect$ or quality).ti.
70. *Patient compliance/ and (improv$ or impact or care or effective or drug therapy or healthcare or medication therapy or prescription? or prescribing or pharmacy service? or quality).ti.
71. ((patient or treatment) adj (adherence or nonadherence or non-adherence or compliance or non-compliance)).ab. or ((patient or treatment) adj2 (adherence or nonadherence or non-adherence or compliance)).ti.
72. or/1-71
73. exp physiotherapist attitude/
74. exp occupational therapist/
75. exp occupational therapist attitude/
76. Physical Therapy Modalities/
77. "Physical Therapy (Specialty)"/
78. (physical therap* or physiotherap*).mp.
79. occupational therapy/ or occupational therap*.mp.
80. Manipulation, Chiropractic/
81. chiropract*.tw.

82. chiroprax*.tw.
83. exp Osteopathic Medicine/
84. exp Manipulation, Osteopathic/
85. OMT.mp.
86. Osteopathic Physician/
87. Osteopathic Medicine/
88. (Osteopath$ adj3 manipulat$).mp.
89. Osteopath*.tw.
90. manual therap*.mp.
91. athletic therap*.mp.
92. sport* therap*.mp.
93. sport* physician*.mp.
94. (Sport* adj1 (medicine or therap*)).tw.
95. Exercise physiolog*.tw.
96. Exercise Therapy/
97. massage therap*.tw.
98. kinesiolog*.tw.
99. Physiatr*.tw.
100. "Physical and Rehabilitation Medicine"/
101. Physical medicine.tw.
102. rehabilitation medicine.tw.
103. orthop?ed*.tw.
104. orthopod*.tw.
105. Doctor of Podiatric Medicine.tw.
106. Podiatry/
107. Podiatric.mp. or podiatrist*.tw.
108. hand therap*.mp.
109. foot therap*.mp.
110. Chiropod*.mp.
111. Acupunctur*.mp.
112. Acupuncture Therapy/
113. Acupressure/
114. or/73-112
115. exp back pain/
116. neck pain/
117. Shoulder pain/
118. Tennis Elbow/
119. exp Tendinopathy/
120. Whiplash Injuries/
121. Sciatica/
122. Intervertebral Disk Displacement/
123. (pain adj3 (neck or back or shoulder? or elbow? or forearm? or wrist? or hand? or arm? or hip? or knee? or ankle? or leg? or foot or feet)).tw.
124. (epicondylitis or tendonitis or tendinitis or bursitis or synovitis or sprain? or strain?).tw.
125. exp Joint Diseases/
126. exp Spinal Diseases/

127. exp Spondylarthritis/
128. exp osteoarthritis/
129. exp Osteoporosis/
130. (arthriti$ or osteoarthriti$ or osteoporo$ or bone loss$).tw.
131. ankle/ or hip/ or knee/
132. elbow/ or wrist/ or shoulder/
133. elbow joint/ or exp hand joints/ or hip joint/ or knee joint/ or sacroiliac joint/ or shoulder joint/
134. exp cervical vertebrae/ or intervertebral disk/ or lumbar vertebrae/ or thoracic vertebrae/
135. exp Back/
136. exp Spine/
137. (spine or spinal).tw.
138. Musculoskeletal Disease*.mp.
139. or/115-138
140. Clinical trial/
141. Randomized controlled trial/
142. Randomization/
143. Single blind procedure/
144. Double blind procedure/
145. Crossover procedure/
146. Placebo/
147. Randomi?ed controlled trial$.tw.
148. Rct.tw.
149. Random allocation.tw.
150. Randomly allocated.tw.
151. Allocated randomly.tw.
152. (allocated adj2 random).tw.
153. Single blind$.tw.
154. Double blind$.tw.
155. ((treble or triple) adj blind$).tw.
156. Placebo$.tw.
157. Prospective study/
158. or/140-157
159. Case study/
160. Case report.tw.
161. Abstract report/ or letter/
162. or/159-161
163. 158 not 162
164. 72 and 114 and 139 and 163

EMBASE search strategy

#	**Query**
S159	S39 AND S61 AND S146 AND S158

S158	S147 or S148 or S149 or S150 or S151 or S152 or S153 or S154 or S155 or S156 or S157
S157	TX allocat* random*
S156	(MH "Quantitative Studies")
S155	(MH "Placebos")
S154	TX placebo*
S153	TX random* allocat*
S152	(MH "Random Assignment")
S151	TX randomi* control* trial*
S150	TX ((singl* n1 blind*) or (singl* n1 mask*)) or TX ((doubl* n1 blind*) or (doubl* n1 mask*)) or TX ((tripl* n1 blind*) or (tripl* n1 mask*)) or TX ((trebl* n1 blind*) or (trebl* n1 mask*))
S149	TX clinic* n1 trial*
S148	PT Clinical trial
S147	(MH "Clinical Trials+")
S146	S62 OR S63 OR S64 OR S70 OR S71 OR S72 OR S73 OR S74 OR S75 OR S76 OR S77 OR S78 OR S79 OR S80 OR S81 OR S82 OR S83 OR S84 OR S85 OR S86 OR S87 OR S88 OR S89 OR S90 OR S91 OR S92 OR S93 OR S94 OR S95 OR S96 OR S97 OR S98 OR S102 OR S103 OR S104 OR S105 OR S106 OR S107 OR S108 OR S111 OR S112 OR S113 OR S114 OR S115 OR S116 OR S117 OR S118 OR S119 OR S120 OR S121 OR S122 OR S123 OR S124 OR S125 OR S126 OR S127 OR S128 OR S131 OR S132 OR S135 OR S136 OR S137 OR S138 OR S139 OR S142 OR S144 OR S145
S145	TI ((therapist N2 initiat*) or (physician N2 initiat*) or (provider N2 initiat*)) or AB ((therapist N2 initiat*) or (physician N2 initiat*) or (provider N2 initiat*))
S144	S140 AND S143
S143	(MH "Health Personnel") OR (MH "Allied Health Personnel") OR (MH "Personnel, Health Facility") or (MH "Physicians")
S142	S140 AND S141
S141	TI ((doctor or physician or practitioner or resident or intern or therapist or allied health or primary care or primary medical care or primary health care or primary patient care or practitioner)) or MW ((doctor or physician or practitioner or resident or intern or therapist or allied health or primary care or primary medical care or primary health care or primary patient care or practitioner))

S140	TI ((dual N2 role) or (enhanced N2 role) or (extend* N2 role)) or AB ((dual N2 role) or (enhanced N2 role) or (extend* N2 role))
S139	AB (doctor N2 role) or (therapist N2 role) or (physician N2 role) or (allied healthcare N2 role)
S138	TI ((leaflet* or pamphlet* or "written information")) or AB ((leaflet* or pamphlet* or "written information"))
S137	TI ((interdisciplinary N1 research) or (collaborative N1 research) or (collaborative N2 care) or (multidisciplinary N2 care) or (interdisciplinary N2 care) OR (collaborative N2 unit) or (multidisciplinary N2 unit) or (interdisciplinary N2 unit)) or AB ((interdisciplinary N1 research) or (collaborative N1 research) or (collaborative N2 care) or (multidisciplinary N2 care) or (interdisciplinary N2 care) OR (collaborative N2 unit) or (multidisciplinary N2 unit) or (interdisciplinary N2 unit))
S136	AB ((patient N1 adherence) or (treatment N1 adherence) or (patient N1 nonadherence) or (treatment N1 nonadherence) or (patient N1 non-adherence) or (treatment N1 non-adherence) or (patient N1 compliance) or (treatment N1 compliance) or (patient N1 non-compliance) or (treatment N1 non-compliance)) or TI ((patient N2 adherence) or (treatment N2 adherence) or (patient N2 nonadherence) or (treatment N2 nonadherence) or (patient N2 non-adherence) or (treatment N2 non-adherence) or (patient N2 compliance) or (treatment N2 compliance))
S135	S133 and S134
S134	TI (improv* or impact or care or effective or healthcare or quality) or MW (improv* or impact or care or effective or healthcare or quality)
S133	(MH "Patient Compliance")
S132	(MH "Telehealth+")
S131	S129 and S130
S130	TI ((impact or improv* or change or changing or effect* or quality)) or MW ((impact or improv* or change or changing or effect* or quality))
S129	TI ((e-health* or e-practice or e-practition* or Tele-diagnos* or tele-consult* or tele-health* or telehealth* or telemonitoring or tele-monitoring or telerehab* or tele-rehab*)) or AB ((e-health* or e-practice or e-practition* or Tele-diagnos* or tele-consult* or tele-health* or telehealth* or telemonitoring or tele-monitoring or telerehab* or tele-rehab*))
S128	TI ((barrier N3 adopt*) or (barrier N3 implement*) or (improv* N3 adopt*) or (improv* N3 implement*) or (increas* N3 adopt*) or (increas* N3 implement*) or (promot* N3 adopt*) or (promot* N3 implement*)) or AB ((barrier N3 adopt*) or (barrier N3 implement*) or (improv* N3 adopt*) or

	(improv* N3 implement*) or (increas* N3 adopt*) or (increas* N3 implement*) or (promot* N3 adopt*) or (promot* N3 implement*))
S127	TI ((barrier or improve or improving or increase or increasing or insurance or insurer or intervention or optim* or promot* or sub-optimal*) and (patient adherence or patient compliance))
S126	AB (barrier N4 patient adherence) or (improve N4 patient adherence) or (improving N4 patient adherence) or (increase N4 patient adherence) or (increasing N4 patient adherence) or (insurance N4 patient adherence) or (insurer N4 patient adherence) or (intervention N4 patient adherence) or (optim* N4 patient adherence) or (promot* N4 patient adherence) or (sub-optimal* N4 patient adherence) or (barrier N4 patient compliance) or (improve N4 patient compliance) or (improving N4 patient compliance) or (increase N4 patient compliance) or (increasing N4 patient compliance) or (insurance N4 patient compliance) or (insurer N4 patient compliance) or (intervention N4 patient compliance) or (optim* N4 patient compliance) or (promot* N4 patient compliance) or (sub-optimal* N4 patient compliance)
S125	TI quality improvement or AB quality improvement
S124	TI ((performance* N2 pay*) or (P4P or "pay for quality improvement?" or P4QI or "fee-for service?" or "payment by result" or capitated or capitation) or (payment N2 mix) or (payment N2 mixed) or (fee N2 mix) or (fee N2 mixed) or (fees N2 mix) or (fees N2 mixed)) or AB ((performance* N2 pay*) or (P4P or "pay for quality improvement?" or P4QI or "fee-for service?" or "payment by result" or capitated or capitation) or (payment N2 mix) or (payment N2 mixed) or (fee N2 mix) or (fee N2 mixed) or (fees N2 mix) or (fees N2 mixed))
S123	TI ((individuali* N2 care) or (individuali* N2 healthcare) or (individuali* N2 medical care) or (integrated N2 care) or (integrated N2 healthcare) or (integrated N2 medical care) or patient-centred or patient-centered or patient-control*) or AB ((individuali* N2 care) or (individuali* N2 healthcare) or (individuali* N2 medical care) or (integrated N2 care) or (integrated N2 healthcare) or (integrated N2 medical care) or patient-centred or patient-centered or patient-control*)
S122	TI ((community and (care or health or healthcare or program* or practice or physician or therapist or allied health or project or doctor))) or AB ((community N2 care) or (community N2 health) or (community N2 healthcare) or (community N2 project) or (community N2 program*) or (community N2 practice) or (community N2 physician) or (community N2 therapist) or (community N2 allied health) or (community N2 doctor)) or TI "community-based"
S121	TI (lay health* worker or community health* worker) or AB (lay health* worker or community health* worker)

S120	TI (self-monitor* or self-care or self-manag* or home-based or home-managed) or AB (self-monitor* or self-care or self-manag* or home-based or home-managed)
S119	TI (guideline adherence or guideline implement* or guideline concordance or AB (guideline adherence or guideline implement* or guideline concordance)
S118	(MH "Guideline Adherence")
S117	TI (health 20 or healthcare 20 or health care 20 or web 20) or AB (health 20 or healthcare 20 or health care 20 or web 20)
S116	TI (blog* or wiki* or PDA or "palm pilot" or blackberr* or Twitter or tweet or tweeting or facebook or social networking or social marketing or youtube) or AB (blog* or wiki* or PDA or "palm pilot" or blackberr* or Twitter or tweet or tweeting or facebook or social networking or social marketing or youtube)
S115	TI skill N2 acquisition or AB skill N2 acquisition
S114	TI (education* or teaching or learning) or MW (education* or teaching or learning) and TI simulat*
S113	TI ((virtual reality or VR Training or VR simulat* or (simulat* N2 skill))) or AB ((virtual reality or VR Training or VR simulat* or (simulat* N2 skill)))
S112	TI ((continuing N2 education*) or ("on the job" N2 education*) or ("off the job" N2 education*) or (postgrad* N2 education*) or (post-grad* N2 education*) or (resident* N2 education*) or (intern N2 education*) or (internship N2 education*) or (workplace N2 education) or (continuing N2 training) or ("on the job" N2 training) or ("off the job" N2 training) or (postgrad* N2 training) or (post-grad* N2 training) or (resident* N2 training) or (intern N2 training) or (internship N2 training) or (workplace N2 training) or (skill education) or (skill training)) or AB ((continuing N2 education*) or ("on the job" N2 education*) or ("off the job" N2 education*) or (postgrad* N2 education*) or (post-grad* N2 education*) or (resident* N2 education*) or (intern N2 education*) or (internship N2 education*) or (workplace N2 education) or (continuing N2 training) or ("on the job" N2 training) or ("off the job" N2 training) or (postgrad* N2 training) or (post-grad* N2 training) or (resident* N2 training) or (intern N2 training) or (internship N2 training) or (workplace N2 training) or (skill education) or (skill training))
S111	S110 and S109
S110	(MH "Education, Continuing")
S109	TI (improv* or impact or care or effective or healthcare) or MW (improv* or impact or care or effective or healthcare)
S108	TI ((Referral and (primary care or primary medical care or primary health care or primary patient care or specialist or allied healthcare or therapist or practitioner or change or changing or improv* or impact or effect* or reduce or

	reducing or increase or increasing or optimi?e or optimal or quality or healthcare or patient care or chronic or management or administration))) or MW ((Referral and (primary care or primary medical care or primary health care or primary patient care or specialist or allied healthcare or therapist or practitioner or change or changing or improv* or impact or effect* or reduce or reducing or increase or increasing or optimi?e or optimal or quality or healthcare or patient care or chronic or management or administration)))
S107	AB referral
S106	TI referral
S105	(MH "Remote Consultation")
S104	(MH "Referral and Consultation")
S103	TI primary care or primary medical care or primary health care or primary patient care or allied healthcare or therapist or practitioner
S102	S99 AND (S100 or S101)
S101	MW (change or changing or improv* or impact or effect* or reduce or reducing or increase or increasing or otimi?e or optimal or quality or healthcare or patient care or chronic or management or administration)
S100	TI (change or changing or improv* or impact or effect* or reduce or reducing or increase or increasing or otimi?e or optimal or quality or healthcare or patient care or chronic or management or administration)
S99	(MH "Practice Patterns") OR (MH "Practice Patterns")
S98	TI (practice pattern or (change N2 practice) or (changing N2 practice)) or AB (practice pattern or (change N2 practice) or (changing N2 practice))
S97	TI ((clinician N2 behavio?r) or (doctor N2 behavio?r) or (therapist N2 behavio?r) or (allied healthcare N2 behavio?r) or (physician N2 behavio?r) or (practitioner N2 behavio?r)) or AB ((doctor N2 behavio?r) or (physician N2 behavio?r) or (practitioner N2 behavio?r))
S96	TI reminder or AB reminder
S95	(MH "Reminder Systems")
S94	TI ("audit and feedback" or (feedback N2 prompt*) or physician feedback or therapist feedback or allied healthcare or doctor feedback or practitioner feedback or provider feedback) or AB ("audit and feedback" or (feedback N2 prompt*) or physician feedback or therapist feedback or allied healthcare or doctor feedback or practitioner feedback or provider feedback)
S93	TI (academic detailing or e-detailing or (opinion N2 leader)) or AB (academic detailing or e-detailing or (opinion N2 leader))

S92	TI ((collaborative or interdisciplin* or inter-disciplin* or multidisciplin* or multi-disciplin* or team or team-based or skill-mix)) or AB ((collaborative or interdisciplin* or inter-disciplin* or multidisciplin* or multi-disciplin* or team or team-based or skill-mix))
S91	TI ((change N2 barrier) or (change N2 faciliat*)) or AB ((change N2 barrier) or (change N2 faciliat*))
S90	TI ((evidence N3 gap) or (identify N3 gap) or (identified N3 gap) or (practice N3 gap)) or AB ((evidence N3 gap) or (identify N3 gap) or (identified N3 gap) or (practice N3 gap))
S89	TI ((computer-tailored N2 feedback) or (individuali?ing N2 feedback) or (individuali?ed N2 feedback) or (personali?e N2 feedback) or (personali?ing N2 feedback) or (tailor* N2 feedback) OR (computer-tailored N2 intervention) or (individuali?ing N2 intervention) or(individuali?ed N2 intervention) or (personali?e N2 intervention) or (personali?ing N2 intervention) or (tailor* N2 intervention) OR (computer-tailored N2 information) or (individuali?ing N2 information) or(individuali?ed N2 information) or (personali?e N2 information) or (personali?ing N2 information) or (tailor* N2 information) OR (computer-tailored N2 plan) or (individuali?ing N2 plan) or(individuali?ed N2 plan) or (personali?e N2 plan) or (personali?ing N2 plan) or (tailor* N2 plan)) or AB ((computer-tailored N2 feedback) or (individuali?ing N2 feedback) or(individuali?ed N2 feedback) or (personali?e N2 feedback) or (personali?ing N2 feedback) or (tailor* N2 feedback) OR (computer-tailored N2 intervention) or (individuali?ing N2 intervention) or(individuali?ed N2 intervention) or (personali?e N2 intervention) or (personali?ing N2 intervention) or (tailor* N2 intervention) OR (computer-tailored N2 information) or (individuali?ing N2 information) or (individuali?ed N2 information) or (personali?e N2 information) or (personali?ing N2 information) or (tailor* N2 information) OR (computer-tailored N2 plan) or (individuali?ing N2 plan) or (individuali?ed N2 plan) or (personali?e N2 plan) or (personali?ing N2 plan) or (tailor* N2 plan))
S88	TI ((health policy or policy maker or decision maker or (policy N3 evidence))) or AB ((health policy or policy maker or decision maker or (policy N3 evidence)))
S87	TI ((evaluat* N2 evidence) or (facilitiat* N2 evidence) or (impact N2 evidence) or (incorporat* N2 evidence) or (policy N2 evidence) or (practice N2 evidence) or (translat* N2 evidence)) or AB ((evaluat* N2 evidence) or (facilitiat* N2 evidence) or (impact N2 evidence) or (incorporat* N2 evidence) or (policy N2 evidence) or (practice N2 evidence) or (translat* N2 evidence))
S86	TI ((research N2 action) or (evidence N2 action)) or AB ((research N2 action) or (evidence N2 action))
S85	TI ((evidence N3 effectiveness) or (intervention N3 effectiveness)) or AB ((evidence N3 effectiveness) or (intervention N3 effectiveness))

S84	TI ((utili?e N2 research) OR (utili?e N2 evidence) or (utili?ing N2 research) OR (utili?ing N2 evidence) OR (implement* N2 research) or (implement* N2 evidence)) or AB ((utili?e N2 research) OR (utili?e N2 evidence) or (utili?ing N2 research) OR (utili?ing N2 evidence) OR (implement* N2 research) or (implement* N2 evidence))
S83	TI ((KT N2 application) or (KT N2 broke*) or (KT N2 diffus*) or(KT N2 disseminat*) or (KT N2 decision*) or (KT N2 exchang*) or (KT N2 implement*) or (KT N2 intervent*) or (KT N2 mobili*) or (KT N2 plan*) or (KT N2 policy) or (KT N2 policies) or (KT N2 strateg*) or (KT N2 translat*) or (KT N2 transfer*) or (KT N2 uptake) or (KT N2 utili*)) or AB ((KT N2 application) or (KT N2 broke*) or (KT N2 diffus*) or(KT N2 disseminat*) or (KT N2 decision*) or (KT N2 exchang*) or (KT N2 implement*) or (KT N2 intervent*) or (KT N2 mobili*) or (KT N2 plan*) or (KT N2 policy) or (KT N2 policies) or (KT N2 strateg*) or (KT N2 translat*) or (KT N2 transfer*) or (KT N2 uptake) or (KT N2 utili*))
S82	TI ((knowledge N2 application) or (knowledge N2 broke*) or (knowledge N2 creation) or (knowledge N2 diffus*) or(knowledge N2 disseminat*) or (knowledge N2 exchang*) or (knowledge N2 implement*) or (knowledge N2 management) or (knowledge N2 mobili*) or (knowledge N2 translat*) or (knowledge N2 transfer*) or (knowledge N2 uptake) or (knowledge N2 utili*) or (evidence* N2 exchang*) or (evidence* N2 translat*) or (evidence* N2 transfer*)) or AB ((knowledge N2 application) or (knowledge N2 broke*) or (knowledge N2 creation) or (knowledge N2 diffus*) or(knowledge N2 disseminat*) or (knowledge N2 exchang*) or (knowledge N2 implement*) or (knowledge N2 management) or (knowledge N2 mobili*) or (knowledge N2 translat*) or (knowledge N2 transfer*) or (knowledge N2 uptake) or (knowledge N2 utili*) or (evidence* N2 exchang*) or (evidence* N2 translat*) or (evidence* N2 transfer*))
S81	TI ((information N2 uptake) or (evidence N2 uptake)) or AB ((information N2 uptake) or (evidence N2 uptake))
S80	TI recommended practice or AB recommended practice
S79	AB ((effectiv* N2 patient-care) or (effectiv* N2 decision*) or (effectiv* N2 doctor) or (effectiv* N2 therapist) or (effectiv* N2 implement*) or (effectiv* N2 health care) or (effectiv* N2 healthcare) or (effectiv* N2 initiative) or (effectiv* N2 management) or (effectiv* N2 practitioner) or (effectiv* N2 provider) or (effectiv* N2 primary care) or (effectiv* N2 primary medical care) or (effectiv* N2 primary health care) or (effectiv* N2 primary patient care)) or AB ((improve* N2 patient-care) or (improve* N2 decision*) or (improve* N2 therapist) or (improve* N2 doctor) or (improve* N2 implement*) or (improve* N2 health care) or (improve* N2 healthcare) or (improve* N2 initiative) or (improve* N2 management) or (improve* N2 practitioner) or (improve* N2 provider) or (improve* N2 primary care) or (improve* N2 primary medical

	care) or (improve* N2 primary health care) or (improve* N2 primary patient care))
S78	TI ((effectiv* N3 adheren*) or (effectiv* N3 care) or (effectiv* N3 therapist) or (effectiv* N3 clinician) or (effectiv* N3 decision*) or (effectiv* N3 doctor) or (effectiv* N3 implement*) or (effectiv* N3 health care) or (effectiv* N3 healthcare) or (effectiv* N3 initiative) or (effectiv* N3 inpatient) or (effectiv* N3 management) or (effectiv* N3 multifacet*) or (effectiv* N3 multi-facet*) or (effectiv* N3 multi-component) or (effectiv* N3 out-patient) or (effectiv* N3 patient-led) or (effectiv* N3 patient-based) or (effectiv* N3 physician) or (effectiv* N3 practi?e) or (effectiv* N3 practitioner) or (effectiv* N3 preventiv*) or (effectiv* N3 preventativ*) or (effectiv* N3 professional) or (effectiv* N3 program) or (effectiv* N3 programme) or (effectiv* N3 provider) or (effectiv* N3 unit) or (effectiv* N3 units)) or TI ((improv* N3 adheren*) or (improv* N3 care) or (improv* N3 clinician) or (improv* N3 decision*) or (improv* N3 doctor) or (improv* N3 implement*) or (improv* N3 health care) or (improv* N3 healthcare) or (improv* N3 initiative) or (improv* N3 inpatient) or (improv* N3 management) or (improv* N3 multifacet*) or (improv* N3 multi-facet*) or (improv* N3 multi-component) or (improv* N3 out-patient) or (improv* N3 patient-led) or (improv* N3 patient-based) or (improv* N3 therapist) or (improv* N3 physician) or (improv* N3 practi?e) or (improv* N3 practitioner) or (improv* N3 preventiv*) or (improv* N3 preventativ*) or (improv* N3 professional) or (improv* N3 program) or (improv* N3 programme) or (improv* N3 provider) or (improv* N3 unit) or (improv* N3 units))
S77	TI ((effectiv* N3 adheren*) or (effectiv* N3 care) or (effectiv* N3 clinician) or (effectiv* N3 decision*) or (effectiv* N3 doctor) or (effectiv* N3 implement*) or (effectiv* N3 health care) or (effectiv* N3 healthcare) or (effectiv* N3 initiative) or (effectiv* N3 inpatient) or (effectiv* N3 management) or (effectiv* N3 multifacet*) or (effectiv* N3 multi facet*) or (effectiv* N3 multi-component) or (effectiv* N3 outpatient) or (effectiv* N3 patient led) or (effectiv* N3 patientbased) or (effectiv* N3 physician) or (effectiv* N3 practi?e) or (effectiv* N3 practitioner) or (effectiv* N3 preventiv*) or (effectiv* N3 preventativ*) or (effectiv* N3 professional) or (effectiv* N3 program) or (effectiv* N3 programme) or (effectiv* N3 provider) or ((effectiv* N3 unit) or (effectiv* N3 units) or (effectiv*N3 therapist) or TI ((improv* N3 adheren*) or (improv* N3 care) or (improv* N3 clinician) or (improv* N3 decision*) or (improv* N3 doctor) or (improv* N3 implement*) or (improv* N3 health care) or (improv* N3 healthcare) or (improv* N3 initiative) or (improv* N3 inpatient) or (improv* N3 management) or (improv* N3 multifacet*) or (improv* N3 multi facet*) or (improv* N3 multicomponent) or (improv* N3 outpatient) or (improv* N3 patient led) or (improv* N3 patient-based) or (improv* N3 physician) or (improv* N3 practi?e) or (improv* N3 practitioner) or (improv* N3 preventiv*) or (improv* N3 preventativ*) or (improv* N3 professional) or (improv* N3 program) or (improv* N3

	programme) or (improv* N3 provider) or (improv* N3 unit) or (improv* N3 units))
S76	(MH "Health Care Delivery")
S75	AB "practice-based" or TI "practice-based"
S74	AB (((evidence N4 intervention) OR (evidence-based N4 intervention) OR evidence-driven)) or TI (((evidence N4 intervention) OR (evidence-based N4 intervention) OR evidence-driven))
S73	AB ((computeri* N2 order N2 system) or (doctor N2 order N2 system) or (medical N2 order N2 system) or (physician N2 order N2 system) or (therapist N2 order N2 system) or (allied healthcare N2 order N2 system) or (practitioner N2 order N2 system) or (provider N2 order N2 system) or (computeri* N2 order N2 entry) or (doctor N2 order N2 entry) or (medical N2 order N2 entry) or (physician N2 order N2 entry) or (practitioner N2 order N2 entry) or (provider N2 order N2 entry) or TI ((computeri* N2 order N2 system) or (doctor N2 order N2 system) or (medical N2 order N2 system) or (physician N2 order N2 system) or (practitioner N2 order N2 system) or (provider N2 order N2 system) or (computeri* N2 order N2 entry) or (doctor N2 order N2 entry) or (medical N2 order N2 entry) or (physician N2 order N2 entry) or (therapist N2 order N2 system) or (allied healthcare N2 order N2 system) or (practitioner N2 order N2 entry) or (provider N2 order N2 entry))
S72	AB (order entry N2 prescription) or (order entry N2 physician) or or (order entry N2 therapist*) or (order entry N2 allied healthcare) or (order entry N2 doctor) or (order entry N2 provider) or (order entry N2 computer*) or (order entry N2 system) or (order entry N2 process*)
S71	TI (order entry)
S70	S68 and S69
S69	S65 or S66 or S67
S68	TI (intervention or improv* or effectiv* or "patient care" or quality or implement*)
S67	(MH "Medical Records+")
S66	(MH "Medical Orders")
S65	(MH "Electronic Order Entry")
S64	TI (integrating or integration)
S63	AB (intervention N3 clinician) or (intervention N3 collaborat*) or (intervention N3 community) or (intervention N3 complex) or (intervention N3 educational) or (intervention N3 doctor) or (intervention N3 allied healthcare) or (intervention N3 therapist*) or (intervention N3 financial) or (intervention N3

	impact) or (intervention N3 individuali?e) or (intervention N3 individuali?ing) or (intervention N3 interdisciplin*) or (intervention N3 multi-component) or (intervention N3 multi-disciplin*) or (intervention N3 multidisciplin*) or (intervention N3 multifacet*) or (intervention N3 multi-facet*) or (intervention N3 organi?ation*) or (intervention N3 personali?e) or (intervention N3 personali?ing) or (intervention N3 physician) or (intervention N3 practitioner) or (intervention N3 prescrib*) or (intervention N3 prescription) or (intervention N3 professional*) or (intervention N3 provider) or (intervention N3 regulatory) or (intervention N3 structured) or (intervention N3 tailor*) or (intervention N3 target*) or (intervention N3 team*)
S62	TI (intervention N3 clinic) or (intervention N3 clinician) or (intervention N3 collaborat*) or (intervention N3 community) or (intervention N3 complex) or (intervention N3 education*) or (intervention N3 effect*) or (intervention N3 financial) or (intervention N3 impact) or (intervention N3 improv*) or (intervention N3 individuali?e) or (intervention N3 individuali?ing) or (intervention N3 inpatient) or (intervention N3 interdisciplin*) or (intervention N3 multi-component) or (intervention N3 multi-disciplin*) or (intervention N3 multidisciplin*) or (intervention N3 multifacet*) or (intervention N3 multi-facet*) or (intervention N3 organi?ation*) or (intervention N3 out-patient) or (intervention N3 personali?e) or (intervention N3 personali?ing) or (intervention N3 physician) or (intervention N3 practitioner) or (intervention N3 allied healthcare) or (intervention N3 therapist*) or (intervention N3 prescrib*) or (intervention N3 prescription) or (intervention N3 professional*) or (intervention N3 provider) or (intervention N3 regulatory) or (intervention N3 structured) or (intervention N3 tailor*) or (intervention N3 target*) or (intervention N3 team*)
S61	S40 OR S41 OR S42 OR S43 OR S44 OR S45 OR S46 OR S47 OR S48 OR S49 OR S50 OR S51 OR S52 OR S53 OR S54 OR S55 OR S56 OR S57 OR S58 OR S59 OR S60
S60	(MH "Podiatry Practice") OR (MH "Podiatr*")
S59	(MH "Acupressure")
S58	(MH "Acupunctur*")
S57	(MH "Foot Therapy")
S56	(MH "Hand Therapy")
S55	(MH "Occupational Medicine")
S54	(MH "Manipulation, Orthopedic")
S53	(MH "Occupational Therapist Attitudes")
S52	(MH "Rehabilitation")
S51	(MH "Physical Medicine") OR (MH "Physical Therapist Attitudes")

S50	(MH "Kinesiolog*")
S49	(MH "Massage Therap*")
S48	(MH "Therapeutic Exercise*")
S47	(MH "Exercise Physiolog*")
S46	(MH "Sports Medicine")
S45	(MH "Physicians, Sports Team")
S44	(MH "Osteopathic Medicine") OR (MH "Manipulation, Osteopathic") OR (MH "Osteopaths")
S43	(MH "Chiropract*")
S42	(MH "Manipulation, Chiropractic") OR (MH "Manual Therapy") OR (MH "Manipulation, Orthopedic")
S41	(MH "Physical Therapy+") OR (MH "Physical Therap*")
S40	(MH "Occupational Therapy+") OR (MH "Occupational Therap*")
S39	S1 OR S2 OR S3 OR S4 OR S5 OR S6 OR S7 OR S8 OR S9 OR S10 OR S11 OR S12 OR S13 OR S14 OR S15 OR S16 OR S17 OR S18 OR S19 OR S20 OR S21 OR S22 OR S23 OR S24 OR S25 OR S26 OR S27 OR S28 OR S29 OR S30 OR S31 OR S32 OR S33 OR S34 OR S35 OR S36 OR S37 OR S38
S38	(MH "Musculoskeletal Diseases")
S37	(MH "Spine")
S36	(MH "Back")
S35	(MH "Intervertebral Disk")
S34	(MH "Cervical Vertebrae") OR (MH "Lumbar Vertebrae") OR (MH "Thoracic Vertebrae") OR (MH "Spondylosis")
S33	(MH "shoulder Joint")
S32	(MH "sacroiliac Joint")
S31	(MH "knee Joint")
S30	(MH "hip Joint")
S29	(MH "hand Joint")
S28	(MH "Elbow Joint")
S27	(MH "shoulder")
S26	(MH "wrist")

S25	(MH "elbow")
S24	(MH "knee")
S23	(MH "hip")
S22	(MH "Ankle")
S21	"bone loss*" OR (MH "Bone Density")
S20	"osteoporo*"
S19	"osteoarthritis*"
S18	(MH "Arthritis")
S17	(MH "Osteoporosis")
S16	(MH "Osteoarthritis")
S15	(MH "Spondylarthritis")
S14	(MH "Spinal Diseases")
S13	(MH "Joint Diseases")
S12	(MH "Sprains and Strains")
S11	(MH "Synovitis")
S10	(MH "Bursitis")
S9	(MH "Tendinopathy")
S8	"epicondylitis"
S7	""pain N3 (neck or back or shoulder? or elbow? or forearm? or wrist? or hand? or arm? or hip? or knee? or ankle? or leg? or foot or feet)""
S6	(MH "Intervertebral Disk Displacement") OR (MH "Intervertebral Disk")
S5	(MH "Sciatica")
S4	(MH "Whiplash Injuries")
S3	(MH "Tennis Elbow")
S2	(MH "Shoulder Pain")
S1	(MH "Low Back Pain") OR (MH "Back Pain") OR (MH "Neck Pain")

COCHRANE search strategy

ID Search

#1 (improv* near/2 practice*):ti,ab

#2 (effectiv* near/2 practice?):ti,ab
#3 (Physician* focused):ti,ab or (multifacet* or multimodal or collaborative*):ti,ab
#4 (intervention* near/2 collaborat*):ti,kw or (intervention* near/2 complex):ti,kw or (intervention* near/2 financial):ti,kw or (intervention* near/2 impact?):ti,kw or (intervention* near/2 individuali*):ti,kw or (intervention* near/2 interdisciplin*):ti,kw or (intervention* near/2 multi-component):ti,kw or (intervention* near/2 multi-disciplin*):ti,kw or (intervention* near/2 multidisciplin*):ti,kw or (intervention* near/2 multifacet*):ti,kw or (intervention* near/2 multi-facet*):ti,kw or (intervention* near/2 organisation*):ti,kw or (intervention* near/2 organiZation*):ti,kw or (intervention* near/2 personali*):ti,kw or (intervention* near/2 physician?):ti,kw or (intervention* near/2 practitioner*):ti,kw or (intervention* near/2 therapist*):ti,kw or (intervention* near/2 allied health care):ti,kw or (intervention* near/2 prescrib*):ti,kw or (intervention* near/2 prescription*):ti,kw or (intervention* near/2 professional*):ti,kw or (intervention* near/2 provider*):ti,kw or (intervention* near/2 regulatory):ti,kw or (intervention* near/2 structured):ti,kw or (intervention* near/2 tailor*):ti,kw or (intervention* near/2 target*):ti,kw or (intervention* near/2 team*):ti,kw
#5 (e-learn* or e-education or e-instruction or elearning or "e learning" or "e train*" or "e curricul*" or "e program*" or m-learn* or virtual reality or VR Training or VR simulat* or Virtual network* or multimedia or multi-media):ti,ab and (physician* or therapist* or allied health or practitioner or clinician* or doctor* or resident or residency or residents or intern or interns):ti
#6 (e-learn* near/2 physician* or e-education near/2 physician* or e-instruction near/2 physician* or elearning near/2 physician* or m-learn* near/2 physician* or virtual reality near/2 physician* or VR Training near/2 physician* or VR simulat* near/2 physician* or Virtual network* near/2 physician*):ti,ab
#7 (booklet* or brochure* or pamphlet* or paper-based or PRINT-BASED or "written material*"):ti,ab
#8 (continuing near/2 education*):ti
#9 inservice:ti,ab or CME:ti,ab or continuing medical education*:ti,ab
#10 (retrain* near/2 DOCTOR* or retrain* near/2 therapist* or retrain* near/2 allied health* or retrain* near/2 HEALTH CARE or retrain* or retrain* near/2 PHYSICIAN* or retrain* near/2 PRACTITIONER* or retrain* near/2 provider*):ti,ab
#11 tailor*:ti,ab or (personali* near/2 patient* personali* near/2 care):ti,ab or (individuali* near/2 patient* individuali* near/2 care):ti,ab
#12 (physician* near/2 behaviour* or physician* near/2 behavior* or therapist* near/2 behaviour* or therapist* near/2 behavior* or allied health* near/2 behaviour* or allied health* near/2 behavior* or provider* near/2 behaviour* or provider* near/2 behavior* or practitioner* near/2 behaviour* or practitioner* near/2 behavior*):ti,ab
#13 referral*:ti,ab
#14 MeSH descriptor Referral and Consultation, this term only
#15 practice pattern*:ti,ab
#16 (organizational near/2 chang* or organisational near/2 chang*):ti,ab
#17 (#1 or #2 or #4 or #5 or #7 or #8 or #9 or #10 or #11 or #12 or #13 or #14 or #15 or #16)
#18 back pain
#19 neck pain
#20 Shoulder pain

#21 Tennis Elbow
#22 Tendinopathy
#23 Whiplash Injuries
#24 Sciatica
#25 Intervertebral Disk Displacement
#26 (pain near/2 (neck or back or shoulder* or elbow* or forearm* or wrist* or hand* or arm* or hip* or knee* or ankle* or leg* or foot or feet)):ti,kw
#27 (epicondylitis or tendonitis or tendinitis or bursitis or synovitis or sprain* or strain*):ti,ab
#28 Joint Diseases
#29 Spinal Diseases
#30 Spondylarthritis
#31 osteoarthritis
#32 Osteoporosis
#33 (arthriti* or osteoarthriti* or osteoporo* or bone loss*):ti,ab
#34 ankle or hip or knee
#35 elbow or wrist or shoulder
#36 elbow joint or exp hand joints or hip joint or knee joint or sacroiliac joint or shoulder joint
#37 cervical vertebrae or intervertebral disk or lumbar vertebrae or thoracic vertebrae
#38 Spine:ti,ab
#39 spinal:ti,ab
#40 musculoskeletal disorder*:ti,kw
#41 (#18 or #19 or #20 or #21 or #22 or #23 or #24 or #25 or #26 or #27 or #28 or #29 or #30 or #31 or #32 or #33 or #34 or #35 or #36 or #37 or #38 or #39 or #40)
#42 Physical Therapy Modalities
#43 "Physical Therapy (Specialty)"
#44 physical therap* or physiotherap*
#45 occupational therap*
#46 Manipulation, Chiropractic
#47 chiropract*
#48 chiroprax*
#49 chiropractic
#50 Osteopathic Medicine
#51 Manipulation, Osteopathic
#52 OMT:ti,kw
#53 Osteopathic Physician*:ti,ab
#54 Osteopathic Medicine*:ti,ab
#55 Osteopath*
#56 manual therap*:ti,ab
#57 athletic therap*:ti,ab
#58 sport* therap*:ti,ab
#59 sport physician*:ti,ab
#60 (Sport* near/2 (medicine or therap*)):ti,ab
#61 Exercise physiolog*:ti,ab
#62 Exercise Therap*:ti,ab
#63 kinesiolog*:ti,ab

#64 Physiatr*:ti,ab
#65 Physical and Rehabilitation Medicine:ti,ab
#66 Physical medicine:ti,ab
#67 rehabilitation medicine:ti,ab
#68 orthoped*:ti,ab
#69 orthopaed*:ti,ab
#70 Podiatr*:ti,ab
#71 hand therap*:ti,ab
#72 foot therap*:ti,ab
#73 Chiropod*:ti,ab
#74 Acupuncture Therap*:ti,ab
#75 Acupunctur*:ti,ab
#76 Acupressure:ti,ab
#77 (#42 or #43 or #44 or #45 or #46 or #47 or #48 or #49 or #50 or #51 or #52 or #53 or #54 or #55 or #56 or #57 or #58 or #59 or #60 or #61 or #63 or #64 or #65 or #66 or #67 or #68 or #70 or #71 or #72 or #73 or #74 or #75 or #76)
#78 (#17 and #41 and #77)
