# Supplementary material for: The effectiveness of interventions designed to increase the uptake of clinical practice guidelines and best practices among musculoskeletal professionals: a systematic review
Source: BMC Health Serv Res. 2018 Jun 8;18:435. doi: 10.1186/s12913-018-3253-0 (PMC5994025; doi:10.1186/s12913-018-3253-0)
Supplement: Supplementary file 2 — Characteristics of excluded studies. (PDF 463 kb) [file 12913_2018_3253_MOESM2_ESM.pdf]

Appendix 2: Characteristics of excluded studies

Study	Reason for exclusion
(Albaladejo, Kovacs et al. 2010)	Not relevant to KT intervention
(Allen, Yancy et al. 2016)	Not relevant to MSK population
(Arroyo-Morales, Cantarero-Villanueva et al. 2012)	Not relevant by population (students)
(Ay, Koldas Dogan et al. 2013)	Not relevant to KT intervention
(Baker, Nelson et al. 2001)	Not relevant to KT intervention
(Barker, Newmany et al. 2013)	Not relevant by population (metal-on-metal hip resurfacing arthroplasty)
(Barry 2003)	Commentary
(Baruth and Wilcox 2011)	Not relevant by study design (pre-post design)
(Bassett and Prapavessis 2007)	Not relevant to KT intervention and population (management of ankle sprain)
(Baxter 2008)	Conference proceeding
(Bekkering, Hendriks et al. 2005)	Multiple publication of already included study
(Bekkering, Hendriks et al. 2005)	Article in Dutch
(Belza, Topolski et al. 2002)	Not relevant to KT intervention
(Ben Salah Frih, Fendri et al. 2009)	Not KT intervention
(Beneciuk and George 2015)	Not RCT
(Bennell, Kyriakides et al. 2014)	Clinical effectiveness
(Bennell, Campbell et al. 2016)	Not relevant by population
(Bennell, Ahamed et al. 2016)	Clinical effectiveness
(Bezalel, Carmeli et al. 2010)	Not relevant to KT intervention
(Bishop, Quon et al. 2010)	Patient directed intervention
(Bozic, Belkora et al. 2013)	For patients with total hip or knee replacement
(Brennan, Fritz et al. 2006)	Not relevant by study design

(Bring, Åsenlöf et al. 2016)	Clinical effectiveness
(Brosseau, Wells et al. 2012)	Patient directed intervention
(Brosseau, Wells et al. 2012)	Patient directed intervention
(Bryant, Lewis et al. 2014)	A description of a KT intervention for ongoing RCT
(Buszewicz, Rait et al. 2006)	Not KT intervention + for GPs
(Cantarero-Villanueva, Fernandez-Lao et al. 2012)	Not relevant by population (students)
(Chaipinyo and Karoonsupcharoen 2009)	Not KT intervention
(Chan, Ford et al. 2015)	Conference proceeding
(Childs, Wu et al. 2014)	cost-effectiveness
(Chipchase and Jull 2015)	Conference proceeding
(Christiansen, Oettingen et al. 2010)	Not relevant by population
(Coleman, Briffa et al. 2008)	Not relevant to KT intervention + protocol
(Cornoiu, Beischer et al. 2011)	Not relevant by population (knee arthroscopy)
(Coudeyre, Givron et al. 2006)	Article in French
(Daltroy, Iversen et al. 1993)	The source is not based on the best evidence
(de Jong, Hopman-Rock et al. 2004)	Patient directed intervention
(Del Pozo-Cruz, Adsuar et al. 2012)	Clinical effectiveness
(Desai, Hughes et al. 2014)	Clinical effectiveness
(Dey, Simpson et al. 2004)	Not relevant by population (GPs)
(Domaille, Mascarenhas et al. 2006)	Not relevant by study design
(Domenech, Sanchez-Zuriaga et al. 2011)	Not relevant by population (students)
(Dziedzic, Stevenson et al. 2009)	Not relevant by study design
(Dziedzic 2015)	Conference proceeding
(Edwards, Jordan et al. 2015)	Conference proceeding

(Engers, Wensing et al. 2005)	Not relevant by population (GPs)
(Eschalier, Descamps et al. 2012)	Not relevant by population (total knee replacement)
(Ettinger, Burns et al. 1997)	Not KT intervention
(Evans, Gilbert et al. 1987)	Not relevant by population (family physician) and intervention
(Feinberg 1992)	Not relevant by population (rheumatoid arthritis)
(Fernandes, Storheim et al. 2010)	Not relevant to KT intervention
(Foster, Nicholls et al. 2015)	Conference proceeding
(Foster, Nicolls et al. 2016)	Conference proceeding
(Gilbey 2011)	Commentary
(Gill and Stella 2013)	Not relevant by study design (retrospective analysis)
(Gohner and Schlicht 2006)	Patient directed intervention
(Gremeaux, Benaïm et al. 2013)	Patient directed intervention
(Gremeaux, Durand et al. 2013)	Pharmacological study
(Gustavsson, Denison et al. 2010)	Not KT intervention
(Gutierrez and Kurlantzick 1997)	Not relevant by study design (pre-post design using claims data)
(Hay, Foster et al. 2006)	Patient directed intervention, not relevant by population (GPs)
(Hazard, Reid et al. 2000)	Patient directed intervention
(Hennig, Haehre et al. 2015)	Conference proceeding
(Heuts, de Bie et al. 2005)	Not KT intervention
(Hill, McPhail et al. 2015)	Clinical effectiveness
(Hoeijenbos, Bekkering et al. 2005)	Cost- effectiveness (economic outcome)
(Hutting, Detaille et al. 2015)	Conference proceeding
(Iles 2013)	Commentary
(Irvine, Russell et al. 2015)	Not for MSK professionals
(Jensen, Jensen et al. 2011)	Not relevant to KT intervention

(Jensen, Bergstrom et al. 2005)	Organisational intervention and its cost-effectiveness analysis
(Jensen, Lundin-Olsson et al. 2002)	Not relevant to KT intervention
(Johnson, Jones et al. 2007)	Patient directed intervention
(Kanemaru, Arahata et al. 2010)	Not relevant by study design (case-control study)
(Karlinsky, Dunn et al. 2006)	Not relevant by study design and targeting physicians
(Karvonen, Paatelma et al. 2015)	Not RCT
(Kearing, Berg et al. 2016)	Patient KT intervention
(Keijsers, Bouter et al. 1992)	The source is neither CPG nor best evidence
(Kendall 2010)	A descriptive report in a conference proceedings
(Ketola, Toivonen et al. 2002)	Not relevant to KT intervention
(Kongsted, Qerama et al. 2008)	Not targeting MSK professional, it was given by trained nurses and recruited from GPs and emergency department.
(Kosterink, Huis In 't Veld et al. 2010)	Not relevant to KT intervention
(Lambeek, Bosmans et al. 2010)	Not relevant to KT intervention + economic evaluation
(Lambeek, van Mechelen et al. 2010)	Not relevant to KT intervention
(Lesnyak, Evstigneeva et al. 2012)	Article in Russian
(Levsen, Hansen et al. 2001)	Not relevant by study design
(Lin, Taylor et al. 2010)	Not relevant by study design (A summary of Cochrane reviews and other evidence and two case-studies)
(Lin, Neoh et al. 2010)	Not relevant to KT intervention
(Linton and Andersson 2000)	Not KT intervention
(Little, Roberts et al. 2001)	Not relevant by population (GPs)
(Lorig, Feigenbaum et al. 1986)	KT intervention targeting patients
(Lysack, Dama et al. 2005)	Targeting post-knee or hip replacement patients

(Macedo, Maher et al. 2012)	It is observational study nested within a RCT, and not KT intervention
(Maitland, Rutten et al. 2010)	Commentary
(Marchiori, Smith et al. 2000)	Conference proceeding
(Marra, Grubisic et al. 2014)	Economic evaluation
(May 2003)	Not relevant by study design (retrospective deign)
(McDonough, Tully et al. 2010)	Protocol
(McGregor, Henley et al. 2012)	Not KT intervention
(Meng, Seekatz et al. 2011)	KT intervention for patients
(Menzel and Robinson 2006)	Not relevant by population (nurses)
(Middleton 2004)	A review
(Miedany, Gaafary et al. 2013)	Not relevant to KT intervention
(Miller, Litva et al. 2009)	Not relevant by study design
(Morris, Morris et al. 2011)	Not relevant by population (discectomy or lateral nerve root decompression surgery) + Cost-effectiveness analysis
(Niedermann, Buchi et al. 2012)	Not relevant by population (rheumatoid arthritis)
(Overmeer, Boersma et al. 2011)	Not relevant by study design (before and after design for therapists)
(Overmeer, Boersma et al. 2009)	Not relevant by study design (before and after design for therapists)
(Pariser, O'Hanlon et al. 2005)	Not relevant to KT intervention
(Parkin-Smith, Norman et al. 2012)	Not relevant to KT intervention
(Perez-Cabezas, Ruiz-Molinero et al. 2015)	Conference proceeding
(Pesco, Chosa et al. 2006)	Not intervention + for students
(Peters, Hentschke et al. 2013)	Article in German
(Pillastrini, Mugnai et al. 2007)	Not KT intervention
(Pisters, Veenhof et al. 2010)	Not KT intervention

(Piyakhachornrot, Aree-Ue et al. 2011)	Not relevant by study design
(Poulsen, Hartvigsen et al. 2013)	Not relevant to KT intervention
(Queally, Kiernan et al. 2013)	Patients were included if they had fracture and the assessment initiation by the participant's general practitioner.
(Rabin 2006)	Not relevant by study design (A case study)
(Rantonen, Vehtari et al. 2014)	Not relevant to KT intervention
(Rasmussen, Holtermann et al. 2013)	Cost-effectiveness
(Rasmussen, Holtermann et al. 2015)	Not KT intervention and not for MSK professionals
(Rasmussen, Holtermann et al. 2016)	Not KT interventions and no MSK professionals
(Richmond, Hansen et al. 2015)	Conference proceeding
(Rose and Probert 2009)	Not relevant by study design and population (GPs)
(Russell, Buttrum et al. 2011)	Total knee arthroplasty
(Saper 2014)	Not relevant to KT intervention
(Saragiotto, de Almeida et al. 2016)	Not RCT
(Schectman, Schroth et al. 2003)	Not relevant by population (internists, family physicians, and associate practitioners (nurse practitioners and physician assistants))
(Schepens, Braun et al. 2012)	Not relevant to KT intervention
(Schneiders, Zusman et al. 1998)	Not relevant to KT intervention
(Scholten-Peeters, Neeleman-van der Steen et al. 2006)	More than 50 % of population are GPs
(Senlof, Denison et al. 2009)	Not relevant to KT intervention
(Shaffer, Brismée et al. 2015)	Not RCT
(Sharma 2016)	Conference proceeding
(Shekelle, Kravitz et al. 2000)	More than 50 % of population are neurologists and general internists

(Shirazi, Wallace et al. 2007)	Not relevant to KT intervention
(Slater, Davies et al. 2015)	Conference proceeding
(Sole, Schneiders et al. 2013)	Not relevant by study design and for students
(Sorensen, Bendix et al. 2010)	Not relevant to KT intervention
(Sparrow, Gottlieb et al. 2011)	Not relevant to KT intervention
(Spink, Menz et al. 2011)	Not relevant to KT intervention
(Stanley, Miller et al. 2001)	Not relevant by study design
(Stevenson, Lewis et al. 2004)	Multiple publication of already included study
(Taheri, Mahdavinejad et al. 2012)	Not relevant to KT intervention
(Tonga, Daskapan et al. 2012)	Article in Turkish
(Tousignant, Moffet et al. 2011)	Not relevant by population (post-knee arthroplasty)
(Tuzun, Akyuz et al. 2013)	Pharmacological treatment
(Van Dillen, Norton et al. 2016)	Clinical effectiveness
(Verhagen, Hupperets et al. 2011)	Not relevant to KT intervention
(Walker 2011)	Review
(Wand, Bird et al. 2004)	Not relevant to KT intervention
(Watson, Wright et al. 2012)	Not relevant by population (students)
(Werner, Storheim et al. 2016)	Clinical and cost- effectiveness
(Willett, Johnson et al. 2011)	Not relevant by study design (pre-experimental design)
(Williams, Bloomfield et al. 2013)	Not relevant to KT intervention
(Williams, Amoakwa et al. 2011)	Feasibility RCT (Pilot).
(Williams, Wiggers et al. 2016)	Conference proceeding

References to studies excluded

Albaladejo, C., F. M. Kovacs, A. Royuela, R. del Pino, J. Zamora and N. Spanish Back Pain Research (2010). "The efficacy of a short education program and a short physiotherapy program for treating low back pain in primary care: a cluster randomized trial." Spine **35**(5): 483-496.
Allen, K. D., W. S. Yancy, Jr., H. B. Bosworth, C. J. Coffman, A. S. Jeffreys, S. K. Datta, J. McDuffie, J. L. Strauss and E. Z. Oddone (2016). "A Combined Patient and Provider Intervention for Management of Osteoarthritis in Veterans: A Randomized Clinical Trial." Annals of Internal Medicine **164**(2): 73-83.
Arroyo-Morales, M., I. Cantarero-Villanueva, C. Fernandez-Lao, M. Guirao-Pineyro, E. Castro-Martin and L. Diaz-Rodriguez (2012). "A blended learning approach to palpation and ultrasound imaging skills through supplementation of traditional classroom teaching with an e-learning package." Manual Therapy **17**(5): 474-478.
Ay, S., S. Koldas Dogan and D. Evcik (2013) "Is there an effective way to prescribe a home-based exercise program in patients with knee osteoarthritis? a randomized controlled study." Turkiye Fiziksel Tip ve Rehabilitasyon Dergisi **59**, 1-6 DOI: 10.4274/tftr.70894.
Baker, K. R., M. E. Nelson, D. T. Felson, J. E. Layne, R. Sarno and R. Roubenoff (2001). "The efficacy of home based progressive strength training in older adults with knee osteoarthritis: a randomized controlled trial." Journal of Rheumatology **28**(7): 1655-1665.
Barker, K., M. Newmany, T. Hughes, A. Kiran, H. Pandit and D. Murray (2013) "Recovery of function following hip resurfacing: A randomised controlled trial comparing a tailored versus standard physiotherapy rehabilitation programme." Osteoarthritis and cartilage **21**, S146-s147.
Barry, H. (2003). "Is physical therapy more effective than guideline-based care for patients with acute low back pain?" Evidence-Based Practice **6**(11): 9, 2p.
Baruth, M. and S. Wilcox (2011). "Effectiveness of two evidence-based programs in participants with arthritis: Findings from the Active for Life Initiative." Arthritis Care & Research **63**(7): 1038-1047.
Bassett, S. F. and H. Prapavessis (2007). "Home-based physical therapy intervention with adherence-enhancing strategies versus clinic-based management for patients with ankle sprains." Physical Therapy **87**(9): 1132-1143.
Baxter, G. D. (2008). "Advice, exercise and opinions in effective management of low back pain: mens sana in corpore sano?" New Zealand Journal of Physiotherapy **36**(2): 78-79.
Bekkering, G. E., H. J. Hendriks, M. W. van Tulder, D. L. Knol, M. Hoeijenbos, R. A. Oostendorp and L. M. Bouter (2005). "Effect on the process of care of an active strategy to implement clinical guidelines on physiotherapy for low back pain: a cluster randomised controlled trial." Quality & Safety in Health Care **14**(2): 107-112.
Bekkering, G. E., H. J. M. Hendriks, M. W. van Tulder, M. A. Koopmanschap, D. L. Knol, R. A. B. Oostendorp and L. M. Bouter (2005). "Effectiveness of an active intervention strategy for the implementation of the Dutch physiotherapy guideline on low back pain [Dutch]." Nederlands Tijdschrift Voor Fysiotherapie **115**(3): 62-67.
Belza, B., T. Topolski, S. Kinne, D. L. Patrick and S. D. Ramsey (2002). "Does adherence make a difference? Results from a community-based aquatic exercise program." Nursing Research **51**(5): 285-291.
Ben Salah Frih, Z., Y. Fendri, A. Jellad, S. Boudoukhane and N. Rejeb (2009). "Efficacy and treatment compliance of a home-based rehabilitation programme for chronic low back pain: a randomized, controlled study." Annals of Physical & Rehabilitation Medicine **52**(6): 485-496.
Beneciuk, J. M. and S. Z. George (2015). "Pragmatic Implementation of a Stratified Primary Care Model for Low Back Pain Management in Outpatient Physical Therapy Settings: Two-Phase, Sequential Preliminary Study." Physical Therapy **95**(8): 1120-1134.
Bennell, K., P. Campbell, T. Egerton, B. Metcalf, J. Kasza, A. Forbes, C. Bills, J. Gale, A. Harris, G. S. Kolt, S. J. Bunker, D. J. Hunter, C. A. Brand and R. S. Hinman (2016). "Telephone coaching to enhance a

physiotherapistprescribed home-based physical activity program for knee osteoarthritis: A randomised clinical trial." Osteoarthritis and Cartilage **24**: S44-S45.
Bennell, K. L., Y. Ahamed, G. Jull, C. Bryant, M. A. Hunt, A. B. Forbes, J. Kasza, M. Akram, B. Metcalf, A. Harris, T. Egerton, J. A. Kenardy, M. K. Nicholas and F. J. Keefe (2016). "Physical Therapist-Delivered Pain Coping Skills Training and Exercise for Knee Osteoarthritis: Randomized Controlled Trial." Arthritis Care and Research **68**(5): 590-602.
Bennell, K. L., M. Kyriakides, P. W. Hodges and R. S. Hinman (2014). "Effects of two physiotherapy booster sessions on outcomes with home exercise in people with knee osteoarthritis: a randomized controlled trial." Arthritis Care & Research **66**(11): 1680-1687.
Bezalel, T., E. Carmeli and M. Katz-Leurer (2010). "The effect of a group education programme on pain and function through knowledge acquisition and home-based exercise among patients with knee osteoarthritis: a parallel randomised single-blind clinical trial." Physiotherapy **96**(2): 137-143.
Bishop, P. B., J. A. Quon, C. G. Fisher and M. F. Dvorak (2010). "The Chiropractic Hospital-based Interventions Research Outcomes (CHIRO) study: a randomized controlled trial on the effectiveness of clinical practice guidelines in the medical and chiropractic management of patients with acute mechanical low back pain." Spine Journal: Official Journal of the North American Spine Society **10**(12): 1055-1064.
Bozic, K. J., J. Belkora, V. Chan, J. Youm, T. Zhou, J. Dupaix, A. N. Bye, C. H. Braddock, 3rd, K. E. Chenok and J. I. Huddleston, 3rd (2013). "Shared decision making in patients with osteoarthritis of the hip and knee: results of a randomized controlled trial." Journal of Bone & Joint Surgery - American Volume **95**(18): 1633-1639.
Brennan, G. P., J. M. Fritz and S. J. Hunter (2006). "Impact of continuing education interventions on clinical outcomes of patients with neck pain who received physical therapy." Physical Therapy **86**(9): 1251-1262.
Bring, A., P. Åsenlöf and A. Söderlund (2016). "What is the comparative effectiveness of current standard treatment, against an individually tailored behavioural programme delivered either on the Internet or face-to-face for people with acute whiplash associated disorder? A randomized controlled trial." Clinical Rehabilitation **30**(5): 441-453.
Brosseau, L., G. A. Wells, G. P. Kenny, R. Reid, A. Maetzel, P. Tugwell, M. Huijbregts, C. McCullough, G. Angelis and L. Chen (2012) "The implementation of a community-based aerobic walking program for mild to moderate knee osteoarthritis: a knowledge translation randomized controlled trial: part II: clinical outcomes." BMC public health **12**, 1073 DOI: 10.1186/1471-2458-12-1073.
Brosseau, L., G. A. Wells, G. P. Kenny, R. Reid, A. Maetzel, P. Tugwell, M. Huijbregts, C. McCullough, G. De Angelis and L. Chen (2012). "The implementation of a community-based aerobic walking program for mild to moderate knee osteoarthritis (OA): a knowledge translation (KT) randomized controlled trial (RCT): Part I: The Uptake of the Ottawa Panel clinical practice guidelines (CPGs)." BMC Public Health **12**: 871-871.
Bryant, C., P. Lewis, K. L. Bennell, Y. Ahamed, D. Crough, G. A. Jull, J. Kenardy, M. K. Nicholas and F. J. Keefe (2014). "Can Physical Therapists Deliver a Pain Coping Skills Program? An Examination of Training Processes and Outcomes." Physical Therapy **94**(10): 1443-1454.
Buszewicz, M., G. Rait, M. Griffin, I. Nazareth, A. Patel, A. Atkinson, J. Barlow and A. Haines (2006) "Self management of arthritis in primary care: randomised controlled trial." BMJ (Clinical research ed.) **333**, 879 DOI: 10.1136/bmj.38965.375718.80.
Cantarero-Villanueva, I., C. Fernandez-Lao, N. Galiano-Castillo, E. Castro-Martin, L. Diaz-Rodriguez and M. Arroyo-Morales (2012). "Evaluation of e-learning as an adjunctive method for the acquisition of skills in bony landmark palpation and muscular ultrasound examination in the lumbopelvic region: a controlled study." Journal of Manipulative & Physiological Therapeutics **35**(9): 727-734.

Chaipinyo, K. and O. Karoonsupcharoen (2009). "No difference between home-based strength training and home-based balance training on pain in patients with knee osteoarthritis: a randomised trial." Australian Journal of Physiotherapy **55**(1): 25-30.
Chan, A., J. Ford, A. Hahne, L. Surkitt, M. Richards, S. Slater, T. Pizzari, M. Davidson, R. Hinman and N. Taylor (2015). "1 year results of a randomised controlled trial comparing subgroup specific physiotherapy against advice for people with low back disorders." Physiotherapy (United Kingdom) **101**: eS207-eS208.
Childs, J. D., S. S. Wu, D. S. Teyhen, M. E. Robinson and S. Z. George (2014) "Prevention of low back pain in the military cluster randomized trial: Effects of brief psychosocial education on total and low back pain-related health care costs." Spine journal **14**, 571-583 DOI: http://dx.doi.org/10.1016/j.spinee.2013.03.019.
Chipchase, L. and G. Jull (2015). "Can a traditional continuing professional development workshop be enhanced?" Physiotherapy (United Kingdom) **101**: eS242.
Christiansen, S., G. Oettingen, B. Dahme and R. Klinger (2010). "A short goal-pursuit intervention to improve physical capacity: a randomized clinical trial in chronic back pain patients." Pain (03043959) **149**(3): 444-452.
Coleman, S., N. K. Briffa, G. Carroll, C. Inderjeeth, N. Cook and J. McQuade (2008). "Effects of self-management, education and specific exercises, delivered by health professionals, in patients with osteoarthritis of the knee." BMC Musculoskeletal Disorders **9**: 133.
Cornoiu, A., A. D. Beischer, L. Donnan, S. Graves and R. de Steiger (2011). "Multimedia patient education to assist the informed consent process for knee arthroscopy." ANZ Journal of Surgery **81**(3): 176-180.
Coudeyre, E., P. Givron, W. Vanbiervliet, C. Benaïm, C. Hérisson, J. Pelissier and S. Poiraudeau (2006) "[The role of an information booklet or oral information about back pain in reducing disability and fear-avoidance beliefs among patients with subacute and chronic low back pain. A randomized controlled trial in a rehabilitation unit]." Annales de réadaptation et de médecine physique : revue scientifique de la Société française de rééducation fonctionnelle de réadaptation et de médecine physique **49**, 600-608 DOI: 10.1016/j.annrmp.2006.05.003.
Daltroy, L. H., M. D. Iversen, M. G. Larson, J. Ryan, C. Zwerling, A. H. Fossel and M. H. Liang (1993). "Teaching and social support: effects on knowledge, attitudes, and behaviors to prevent low back injuries in industry." Health Education Quarterly **20**(1): 43-62.
de Jong, O. R. W., M. Hopman-Rock, E. C. M. Tak and N. S. Klazinga (2004). "An implementation study of two evidence-based exercise and health education programmes for older adults with osteoarthritis of the knee and hip." Health Education Research **19**(3): 316-325.
Del Pozo-Cruz, B., J. C. Adsuar, J. Parraca, J. s. Del Pozo-Cruz, A. Moreno and N. Gusi (2012). "A Web-Based Intervention to Improve and Prevent Low Back Pain Among Office Workers : A Randomized Controlled Trial." Journal of Orthopaedic & Sports Physical Therapy **42**(10): 831-841.
Desai, P. M., S. L. Hughes, K. E. Peters and R. J. Mermelstein (2014) "Impact of telephone reinforcement and negotiated contracts on behavioral predictors of exercise maintenance in older adults with osteoarthritis." American journal of health behavior **38**, 465-477 DOI: http://dx.doi.org/10.5993/AJHB.38.3.15.
Dey, P., C. W. Simpson, S. I. Collins, G. Hodgson, C. F. Dowrick, A. J. Simison and M. J. Rose (2004) "Implementation of RCGP guidelines for acute low back pain: a cluster randomised controlled trial." British journal of general practice **54**, 33-37.
Domaille, M., R. Mascarenhas, N. Dayal and J. Kirwan (2006). "Evaluation of the Bristol Royal Infirmary physiotherapy programme for the management of patients with osteoarthritis of the knee." Musculoskeletal Care **4**(2): 78-87.

Domenech, J., D. Sanchez-Zuriaga, E. Segura-Orti, B. Espejo-Tort and J. F. Lison (2011). "Impact of biomedical and biopsychosocial training sessions on the attitudes, beliefs, and recommendations of health care providers about low back pain: a randomised clinical trial." Pain **152**(11): 2557-2563.
Dziedzic, K. (2015). "Implementing osteoarthritis guidelines in UK primary care: Mosaics cluster randomised controlled trial." Osteoarthritis and Cartilage **23**: A30.
Dziedzic, K., K. Stevenson, E. Thomas, J. Sim and E. Hay (2009). "Development and implementation of a physiotherapy intervention for use in a pragmatic randomized controlled trial in primary care for shoulder pain." Musculoskeletal Care **7**(2): 67-77.
Edwards, J. J., K. P. Jordan, M. Porcheret, E. L. Healey, C. Jinks, J. Bedson, K. Clarkson, E. M. Hay and K. S. Dziedzic (2015). "Effect of a model consultation on quality of care of osteoarthritis: A primary care cluster randomised trial." Annals of the Rheumatic Diseases **74**: 108-109.
Engers, A. J., M. Wensing, M. W. van Tulder, A. Timmermans, R. A. Oostendorp, B. W. Koes and R. Grol (2005). "Implementation of the Dutch low back pain guideline for general practitioners: a cluster randomized controlled trial." Spine **30**(6): 559-600.
Eschalier, B., S. Descamps, B. Pereira, M. G. Girard, S. Boisgard and E. Coudeyre (2012) "Evaluation of a pre operative education approach for patient undergoing total knee replacement, Evaluation d'une demarche d'education therapeutique preoperatoire avant arthroplastie totale de genou. [French, English]." Annals of physical and rehabilitation medicine **55**, e117-e118+e120 DOI: http://dx.doi.org/10.1016/j.rehab.2012.07.314.
Ettinger, W. H., Jr., R. Burns, S. P. Messier, W. Applegate, W. J. Rejeski, T. Morgan, S. Shumaker, M. J. Berry, M. O'Toole, J. Monu and T. Craven (1997). "A randomized trial comparing aerobic exercise and resistance exercise with a health education program in older adults with knee osteoarthritis: The Fitness Arthritis and Seniors Trial (FAST)." JAMA: Journal of the American Medical Association **277**(1): 25-31.
Evans, C., J. R. Gilbert, W. Taylor and A. Hildebrand (1987). "A randomized controlled trial of flexion exercises, education, and bed rest for patients with acute low back pain." Physiotherapy Canada **39**(2): 96-101.
Feinberg, J. (1992). "Effect of the arthritis health professional on compliance with use of resting hand splints by patients with rheumatoid arthritis." Arthritis Care & Research **5**(1): 17-23.
Fernandes, L., K. Storheim, L. Sandvik, L. Nordsletten and M. A. Risberg (2010). "Efficacy of patient education and supervised exercise vs patient education alone in patients with hip osteoarthritis: a single blind randomized clinical trial." Osteoarthritis & Cartilage **18**(10): 1237-1243.
Foster, N., E. Nicholls, M. Holden, E. L. Healey, J. Kigozi, S. Jowett, S. Tooth and E. M. Hay (2015). "Improving the effectiveness of exercise therapy for older adults with knee osteoarthritis: A pragmatic randomised controlled trial (the beep trial)." Annals of the Rheumatic Diseases **74**: 108.
Foster, N. E., E. Nicolls, M. A. Holden, E. L. Healey, S. Tooth, J. Kigozi, S. Jowett and E. M. Hay (2016). "Improving the effectiveness of exercise therapy for older adults with knee pain: A pragmatic randomised controlled trial (the beep trial)." Osteoarthritis and Cartilage **24**: S43-S44.
Gilbey, A. (2011). "Evidence-based clinical practice guidelines vs. family physician usual care for the treatment of acute lower back pain: what is the role of chiropractic?" Focus on Alternative & Complementary Therapies **16**(3): 243-245.
Gill, S. D. and J. Stella (2013). "Implementation and performance evaluation of an emergency department primary practitioner physiotherapy service for patients with musculoskeletal conditions." Emergency Medicine Australasia **25**(6): 558-564.
Gohner, W. and W. Schlicht (2006). "Preventing chronic back pain: evaluation of a theory-based cognitive-behavioural training programme for patients with subacute back pain." Patient Education & Counseling **64**(1-3): 87-95.

Gremeaux, V., C. Benaïm, S. Poiraudeau, C. Hérisson, A. Dupeyron and E. Coudeyre (2013). "Evaluation of the benefits of low back pain patients' education workshops during spa therapy." Joint Bone Spine **80**(1): 82-87.
Gremeaux, V., S. Durand, C. Benaim, C. Herisson, J. Monleaud, S. Hansel and E. Coudeyre (2013). "Evaluation of various ways to deliver information concerning non-steroidal anti-inflammatory drugs to osteoarthritis patients." Annals of Physical & Rehabilitation Medicine **56**(1): 14-29.
Gustavsson, C., E. Denison and L. von Koch (2010). "Self-management of persistent neck pain: a randomized controlled trial of a multi-component group intervention in primary health care." European Journal of Pain **14**(6): 630.e631-630.e611.
Gutierrez, B. and V. Kurlantzick (1997) "Using low back pain guidelines to change health care professional practice patterns [abstract]." Abstract Book/Association for Health Services Research **14**, 105-106.
Hay, E. M., N. E. Foster, E. Thomas, G. Peat, M. Phelan, H. E. Yates, A. Blenkinsopp and J. Sim (2006). "Effectiveness of community physiotherapy and enhanced pharmacy review for knee pain in people aged over 55 presenting to primary care: pragmatic randomised trial." BMJ **333**(7576): 995.
Hazard, R. G., S. Reid, L. D. Haugh and G. McFarlane (2000) "A controlled trial of an educational pamphlet to prevent disability after occupational low back injury." Spine **25**, 1419-1423.
Hennig, T., L. Haehre, V. T. Hornburg, P. Mowinckel, E. S. Norli and I. Kjeken (2015). "Effect of home-based hand exercises in women with hand osteoarthritis: A randomised controlled trial." Annals of the Rheumatic Diseases **74**(8): 1501-1508.
Heuts, P. H., R. de Bie, M. Drietelaar, K. Aretz, M. Hopman-Rock, C. H. Bastiaenen, J. F. Metsemakers, C. van Weel and O. van Schayck (2005). "Self-management in osteoarthritis of hip or knee: a randomized clinical trial in a primary healthcare setting." Journal of Rheumatology **32**(3): 543-549.
Hill, A. M., S. M. McPhail, N. Waldron, C. Etherton-Beer, K. Ingram, L. Flicker, M. Bulsara and T. P. Haines (2015) "Fall rates in hospital rehabilitation units after individualised patient and staff education programmes: a pragmatic, stepped-wedge, cluster-randomised controlled trial." Lancet (London, England) **385**, 2592-2599 DOI: 10.1016/S0140-6736(14)61945-0.
Hoeijenbos, M., T. Bekkering, L. Lamers, E. Hendriks, M. van Tulder and M. Koopmanschap (2005). "Cost-effectiveness of an active implementation strategy for the Dutch physiotherapy guideline for low back pain." Health Policy **75**(1): 85-98.
Hutting, N., S. I. Detaille, Y. F. Heerkens, J. A. Engels, J. B. Staal and M. W. G. Nijhuis-Van Der Sanden (2015). "Experiences of participants in a self-management program for employees suffering from complaints of the arm, neck or shoulder (CANS)." Physiotherapy (United Kingdom) **101**: eS619.
Iles, R. (2013). "A physiotherapy telephone assessment and advice service for patients with musculoskeletal problems can improve the process of care while maintaining clinical effectiveness." Journal of Physiotherapy (Elsevier) **59**(2): 130-130.
Irvine, A. B., H. Russell, M. Manocchia, D. E. Mino, T. Cox Glassen, R. Morgan, J. M. Gau, A. J. Birney and D. V. Ary (2015) "Mobile-Web app to self-manage low back pain: randomized controlled trial." Journal of medical Internet research **17**, e1 DOI: 10.2196/jmir.3130.
Jensen, C., O. K. Jensen, D. H. Christiansen and C. V. Nielsen (2011) "One-year follow-up in employees sick-listed because of low back pain: randomized clinical trial comparing multidisciplinary and brief intervention." Spine **36**, 1180-1189 DOI: 10.1097/BRS.0b013e3181eba711.
Jensen, I. B., G. Bergstrom, T. Ljungquist and L. Bodin (2005). "A 3-year follow-up of a multidisciplinary rehabilitation programme for back and neck pain." Pain **115**(3): 273-283.
Jensen, J., L. Lundin-Olsson, L. Nyberg and Y. Gustafson (2002) "Fall and injury prevention in older people living in residential care facilities. A cluster randomized trial." Annals of internal medicine **136**, 733-741.

Johnson, R. E., G. T. Jones, N. J. Wiles, C. Chaddock, R. G. Potter, C. Roberts, D. P. Symmons, P. J. Watson, D. J. Torgerson and G. J. Macfarlane (2007). "Active exercise, education, and cognitive behavioral therapy for persistent disabling low back pain: a randomized controlled trial." Spine **32**(15): 1578-1585.
Kanemaru, A., K. Arahata, T. Ohta, T. Katoh, H. Tobimatsu and T. Horiuchi (2010). "The efficacy of home-based muscle training for the elderly osteoporotic women: The effects of daily muscle training on quality of life (QoL)." Archives of Gerontology & Geriatrics **51**(2): 169-172.
Karlinsky, H., C. Dunn, B. Clifford, J. Atkins, G. Pachev, K. Cunningham, P. Fenrich and Y. Bayani (2006) "Workplace injury management: using new technology to deliver and evaluate physician continuing medical education." Journal of occupational rehabilitation **16**, 719-730 DOI: 10.1007/s10926-006-9047-y.
Karvonen, E., M. Paatelma, J.-P. Kesonen and A. O. Heinonen (2015). "Knowledge translation from continuing education to physiotherapy practice in classifying patients with low back pain." Journal of Manual & Manipulative Therapy (Maney Publishing) **23**(2): 68-74.
Kearing, S., S. Z. Berg and J. D. Lurie (2016). "Can decision support help patients with spinal stenosis make a treatment choice?: A prospective study assessing the impact of a patient decision aid and health coaching." Spine **41**(7): 563-567.
Keijsers, J. F. E., L. M. Bouter, R. M. Meertens, A. G. H. Kessels and P. G. Knipschild (1992). "The impact of back school research on the beliefs of health care professionals: a randomised survey of general practitioners and physiotherapists." Physiotherapy Theory & Practice **8**(2): 79-83.
Kendall, N. (2010). "Back Up -- a UK example of evidence-informed back and neck pain management." New Zealand Journal of Physiotherapy **38**(2): 70-70.
Ketola, R., R. Toivonen, M. Hakkanen, R. Luukkonen, E. P. Takala, E. Viikari-Juntura and E. Expert Group in (2002). "Effects of ergonomic intervention in work with video display units." Scandinavian Journal of Work, Environment & Health **28**(1): 18-24.
Kongsted, A., E. Qerama, H. Kasch, F. W. Bach, L. Korsholm, T. S. Jensen and T. Bendix (2008) "Education of patients after whiplash injury: is oral advice any better than a pamphlet?" Spine **33**, E843-848 DOI: 10.1097/BRS.0b013e318182bee2.
Kosterink, S. M., R. M. Huis In 't Veld, B. Cagnie, M. Hasenbring and M. M. Vollenbroek-Hutten (2010). "The clinical effectiveness of a myofeedback-based teletreatment service in patients with non-specific neck and shoulder pain: a randomized controlled trial." Journal of Telemedicine & Telecare **16**(6): 316-321.
Lambeek, L. C., J. E. Bosmans, B. J. Van Royen, M. W. Van Tulder, W. Van Mechelen and J. R. Anema (2010). "Effect of integrated care for sick listed patients with chronic low back pain: economic evaluation alongside a randomised controlled trial." BMJ **341**: c6414.
Lambeek, L. C., W. van Mechelen, D. L. Knol, P. Loisel and J. R. Anema (2010). "Randomised controlled trial of integrated care to reduce disability from chronic low back pain in working and private life." BMJ **340**: c1035.
Lesnyak, O. M., L. P. Evstigneeva, N. Kuznetsova, A. Vorobyeva, J. Safonova, S. Bulgakova, M. Kirpikova, M. Strunina, M. Telushenko, M. Nekrasova and O. Nesmeyanova (2012) "Multicenter randomized trial of interactive educational program in patients with osteoporosis." Osteoporosis international **23**, S232-s233 DOI: http://dx.doi.org/10.1007/s00198-012-1928-7.
Levsen, M. L., M. L. Hansen, A. D. Kent, J. J. Sieren, J. P. Thoreson and K. P. Farrell (2001). "Effects of physical therapist training on outcomes of patients with chronic low back pain or chronic shoulder pain." Journal of Manual & Manipulative Therapy (Journal of Manual & Manipulative Therapy) **9**(2): 84-91.
Lin, C. C., D. Taylor, S. M. A. Bierma-Zeinstra and C. G. Maher (2010). "Linking evidence and practice. Exercise for osteoarthritis of the knee." Physical Therapy **90**(6): 839-842.
Lin, S., C. Neoh, Y. Huang, K. Wang, H. Ng and H. Shi (2010). "Educational program for myofascial pain syndrome." Journal of Alternative & Complementary Medicine **16**(6): 633-640.

Linton, S. J. and T. Andersson (2000). "Can chronic disability be prevented? A randomized trial of a cognitive-behavior intervention and two forms of information for patients with spinal pain." Spine **25**(21): 2825-2831; discussion 2824.
Little, P., L. Roberts, H. Blowers, J. Garwood, T. Cantrell, J. Langridge and J. Chapman (2001). "Should we give detailed advice and information booklets to patients with back pain? A randomized controlled factorial trial of a self-management booklet and doctor advice to take exercise for back pain... including commentary by Thomas E." Spine **26**(19): 2065-2072.
Lorig, K., P. Feigenbaum, C. Regan, E. Ung, R. L. Chastain and H. R. Holman (1986). "A comparison of lay-taught and professional-taught arthritis self-management courses." Journal of Rheumatology **13**(4): 763-767.
Lysack, C., M. Dama, S. Neufeld and E. Andreassi (2005) "A compliance and satisfaction with home exercise: a comparison of computer-assisted video instruction and routine rehabilitation practice." Journal of allied health **34**, 76-82.
Macedo, L. G., C. G. Maher, J. Latimer and J. H. McAuley (2012). "Feasibility of using short message service to collect pain outcomes in a low back pain clinical trial." Spine **37**(13): 1151-1155.
Maitland, M., G. M. Rutten, J. Harting and E. J. Hendriks (2010). "On "adherence to clinical practice guidelines..."." Physical Therapy **90**(12): 1899-1900.
Marchiori, D. M., M. Smith and I. D. McLean (2000) "An evidence-based interactive seminar approach to guiding clinician decision-making for ordering diagnostic radiographs in a chiropractic educational institution." **16**, 377.
Marra, C. A., M. Grubisic, J. Cibere, K. A. Grindrod, J. C. Woolcott, L. Gastonguay and J. M. Esdaile (2014) "Cost-utility analysis of a multidisciplinary strategy to manage osteoarthritis of the knee: Economic evaluation of a cluster randomized controlled trial study." Arthritis care & research **66**, 810-816 DOI: http://dx.doi.org/10.1002/acr.22232.
May, S. (2003). "An outcome audit for musculoskeletal patients in primary care." Physiotherapy Theory & Practice **19**(4): 189-198.
McDonough, S. M., M. A. Tully, S. R. O'Connor, A. Boyd, D. P. Kerr, S. M. O'Neill, A. Delitto, I. Bradbury, C. Tudor-Locke, D. G. Baxter and D. A. Hurley (2010). "The Back 2 Activity Trial: education and advice versus education and advice plus a structured walking programme for chronic low back pain." BMC Musculoskeletal Disorders **11**: 163-163.
McGregor, A. H., A. Henley, T. P. Morris, C. J. Dore and F. team (2012). "An evaluation of a postoperative rehabilitation program after spinal surgery and its impact on outcome." Spine **37**(7): E417-422.
Meng, K., B. Seekatz, H. Roband, U. Worringen, H. Vogel and H. Faller (2011). "Intermediate and long-term effects of a standardized back school for inpatient orthopedic rehabilitation on illness knowledge and self-management behaviors: a randomized controlled trial." Clinical Journal of Pain **27**(3): 248-257.
Menzel, N. N. and M. E. Robinson (2006) "Back pain in direct patient care providers: early intervention with cognitive behavioral therapy." Pain management nursing **7**, 53-63 DOI: 10.1016/j.pmn.2006.02.002.
Middleton, A. (2004). "Chronic low back pain: patient compliance with physiotherapy advice and exercise, perceived barriers and motivation." Physical Therapy Reviews **9**(3): 153-160.
Miedany, Y., M. Gaafary, S. Youssef, N. Aroussy, I. Ahmed and D. Palmer (2013) "A step forward to close the loop: Applying "joint-fitness" and patient reported outcome measures to tailor a patient-specific education program." Annals of the Rheumatic Disease **71** DOI: http://dx.doi.org/10.1136/annrheumdis-2012-eular.345.
Miller, J. S., A. Litva and M. Gabbay (2009). "Motivating patients with shoulder and back pain to self-care: can a videotape of exercise support physiotherapy?" Physiotherapy **95**(1): 29-35.
Morris, S., T. P. Morris, A. H. McGregor, C. J. Dore and K. Jamrozik (2011) "Function after spinal treatment, exercise, and rehabilitation: Cost-effectiveness analysis based on a randomized controlled trial." Spine **36**, 1807-1814 DOI: http://dx.doi.org/10.1097/BRS.0b013e31821cba1f.

Niedermann, K., S. Buchi, A. Ciurea, R. Kubli, C. Steurer-Stey, P. M. Villiger and R. A. De Bie (2012). "Six and 12 months' effects of individual joint protection education in people with rheumatoid arthritis: a randomized controlled trial." Scandinavian Journal of Occupational Therapy **19**(4): 360-369.
Overmeer, T., K. Boersma, E. Denison and S. J. Linton (2011). "Does Teaching Physical Therapists to Deliver a Biopsychosocial Treatment Program Result in Better Patient Outcomes? A Randomized Controlled Trial." Physical Therapy **91**(5): 804-819.
Overmeer, T., K. Boersma, C. J. Main and S. J. Linton (2009). "Do physical therapists change their beliefs, attitudes, knowledge, skills and behaviour after a biopsychosocially orientated university course?" Journal of Evaluation in Clinical Practice **15**(4): 724-732.
Pariser, D., A. O'Hanlon and L. Espinoza (2005). "Effects of telephone intervention on arthritis self-efficacy, depression, pain, and fatigue in older adults with arthritis." Journal of Geriatric Physical Therapy **28**(3): 67-73.
Parkin-Smith, G. F., I. J. Norman, E. Briggs, E. Angier, T. G. Wood and J. W. Brantingham (2012). "A structured protocol of evidence-based conservative care compared with usual care for acute nonspecific low back pain: a randomized clinical trial." Archives of Physical Medicine & Rehabilitation **93**(1): 11-20.
Perez-Cabezas, V., C. Ruiz-Molinero, R. Chillon-Martinez, J. Aguilera-Santacruz, J. J. Jimenez-Rejano and M. J. Vinolo-Gil (2015). "Effectiveness of a program of reeducation oculo-cervical procedures for chronic neck pain." Physiotherapy (United Kingdom) **101**: eS38.
Pesco, M. S., E. Chosa and N. Tajima (2006). "Comparative study of hands-on therapy with active exercises vs education with active exercises for the management of upper back pain." Journal of Manipulative & Physiological Therapeutics **29**(3): 228-235.
Peters, S., C. Hentschke and K. Pfeifer (2013). "Internet-based "e-Training" as Exercise Intervention for Health Promotion: Results from 2 Intervention Studies] [German]." Rehabilitation **52**(3): 173-181.
Pillastrini, P., R. Mugnai, C. Farneti, L. Bertozzi, R. Bonfiglioli, S. Curti, S. Mattioli and F. S. Violante (2007). "Evaluation of two preventive interventions for reducing musculoskeletal complaints in operators of video display terminals." Physical Therapy **87**(5): 536-544.
Pisters, M. F., C. Veenhof, D. H. de Bakker, F. G. Schellevis and J. Dekker (2010). "Behavioural graded activity results in better exercise adherence and more physical activity than usual care in people with osteoarthritis: a cluster-randomised trial." Journal of Physiotherapy **56**(1): 41-47.
Piyakhachornrot, N., S. Aree-Ue, P. Putwatana and V. Kawinwonggowit (2011). "Impact of an integerated health education and exercise program in middle-aged Thai adults with osteoarthritis of the knee." Orthopaedic Nursing **30**(2): 134-142.
Poulsen, E., J. Hartvigsen, H. W. Christensen, E. M. Roos, W. Vach and S. Overgaard (2013). "Patient education with or without manual therapy compared to a control group in patients with osteoarthritis of the hip. A proof-of-principle three-arm parallel group randomized clinical trial." Osteoarthritis & Cartilage **21**(10): 1494-1503.
Queally, J. M., C. Kiernan, M. Shaikh, F. Rowan and D. Bennett (2013). "Initiation of osteoporosis assessment in the fracture clinic results in improved osteoporosis management: a randomised controlled trial." Osteoporosis International **24**(3): 1089-1094.
Rabin, A. (2006). "Evidence in practice... evidence to support the use of eccentric strengthening exercises to decrease pain and increase function in patients with patellar tendinopathy?" Physical Therapy **86**(3): 450-456.
Rantonen, J., A. Vehtari, J. Karppinen, S. Luoto, E. Viikari-Juntura, M. Hupli, A. Malmivaara and S. Taimela (2014) "Face-to-face information combined with a booklet versus a booklet alone for treatment of mild low-back pain: A randomized controlled trial." Scandinavian journal of work, environment & health **40**, 156-166 DOI: http://dx.doi.org/10.5271/sjweh.3398.

Rasmussen, C. D., A. Holtermann, H. Bay, K. Sogaard and M. Birk Jorgensen (2015). "A multifaceted workplace intervention for low back pain in nurses' aides: a pragmatic stepped wedge cluster randomised controlled trial." Pain **156**(9): 1786-1794.
Rasmussen, C. D., A. Holtermann, O. S. Mortensen, K. Sogaard and M. B. Jorgensen (2013). "Prevention of low back pain and its consequences among nurses' aides in elderly care: a stepped-wedge multi-faceted cluster-randomized controlled trial." BMC Public Health **13**: 1088.
Rasmussen, C. D. N., A. Holtermann, M. B. Jørgensen, A. Ørberg, O. S. Mortensen and K. Søgaard (2016). "A multi-faceted workplace intervention targeting low back pain was effective for physical work demands and maladaptive pain behaviours, but not for work ability and sickness absence: Stepped wedge cluster randomised trial." Scandinavian Journal of Public Health **44**(6): 560-570.
Richmond, H., Z. Hansen, D. Davies, E. Williamson and S. Lamb (2015). "Implementation of the best intervention: A group cognitive behavioural approach for patients with low back pain." Physiotherapy (United Kingdom) **101**: eS815-eS816.
Rose, R. and S. Probert (2009). "Development and implementation of a hand therapy extended scope practitioner clinic to support the 18-week waiting list initiative." Hand Therapy **14**(4): 95-104.
Russell, T. G., P. Buttrum, R. Wootton and G. A. Jull (2011). "Internet-based outpatient telerehabilitation for patients following total knee arthroplasty: a randomized controlled trial." Journal of Bone & Joint Surgery, American Volume **93**(2): 113-120.
Saper, R. (2014). "Predictors of Adherence to Treatment for Chronic Low Back Pain in a 12-week RCT Comparing Yoga, Physical Therapy, and Education." Journal of Alternative & Complementary Medicine **20**(5): A56-A56.
Saragiotto, B. T., M. O. de Almeida, T. P. Yamato and C. G. Maher (2016). "Multidisciplinary Biopsychosocial Rehabilitation for Nonspecific Chronic Low Back Pain." Physical Therapy **96**(6): 759-763.
Schectman, J. M., W. S. Schroth, D. Verme and J. D. Voss (2003). "Randomized controlled trial of education and feedback for implementation of guidelines for acute low back pain." Journal of General Internal Medicine **18**(10): 773-780.
Schepens, S. L., M. E. Braun and S. L. Murphy (2012) "Effect of tailored activity pacing on self-perceived joint stiffness in adults with knee or hip osteoarthritis." The American journal of occupational therapy : official publication of the American Occupational Therapy Association **66**, 363-367 DOI: 10.5014/ajot.2010.004036.
Schneiders, A. G., M. Zusman and K. P. Singer (1998). "Exercise therapy compliance in acute low back pain patients." Manual Therapy **3**(3): 147-152.
Scholten-Peeters, G. G. M., C. W. M. Neeleman-van der Steen, D. A. W. van der Windt, E. J. M. Hendriks, A. P. Verhagen and R. A. B. Oostendorp (2006). "Education by general practitioners or education and exercises by physiotherapists for patients with whiplash-associated disorders? A randomized clinical trial." Spine **31**(7): 723-731.
Senlof, P., E. Denison and P. Lindberg (2009). "Long-term follow-up of tailored behavioural treatment and exercise based physical therapy in persistent musculoskeletal pain: A randomized controlled trial in primary care." European Journal of Pain **13**(10): 1080-1088.
Shaffer, S. M., J.-M. Brismée, C. A. Courtney and P. S. Sizer (2015). "The status of temporomandibular and cervical spine education in credentialed orthopedic manual physical therapy fellowship programs: a comparison of didactic and clinical education exposure." Journal of Manual & Manipulative Therapy (Maney Publishing) **23**(1): 51-56.
Sharma, R. (2016). "Effectiveness of Educational and Selected Exercise Programme to Reduce Back Pain in Staff Nurses." International Journal of Nursing Education **8**(2): 62-67.
Shekelle, P. G., R. L. Kravitz, J. Beart, M. Marger, M. Wang and M. Lee (2000). "Are nonspecific practice guidelines potentially harmful? A randomized comparison of the effect of nonspecific versus specific guidelines on physician decision making." Health Services Research **34**(7): 1429-1448.

Shirazi, K. K., L. M. Wallace, S. Niknami, A. Hidarnia, G. Torkaman, M. Gilchrist and S. Faghihzadeh (2007). "A home-based, transtheoretical change model designed strength training intervention to increase exercise to prevent osteoporosis in Iranian women aged 40-65 years: a randomized controlled trial." Health Education Research **22**(3): 305-317.
Slater, H., S. Davies, G. Milne, J. Kelso, M. Slattery and A. Briggs (2015). "The painhealth website: A western australian policy-into-practice initiative to deliver holistic, consumer-focused best-evidence pain management for people with musculoskeletal pain." Physiotherapy (United Kingdom) **101**: eS1410.
Sole, G., A. Schneiders, K. Hébert-Losier and M. Perry (2013). "Perceptions by physio-theratoy students and faculty staff of a multimedia learning resource for musculoskeletal practical skills teaching." New Zealand Journal of Physiotherapy **41**(2): 58-64.
Sorensen, P. H., T. Bendix, C. Manniche, L. Korsholm, D. Lemvigh and A. Indahl (2010). "An educational approach based on a non-injury model compared with individual symptom-based physical training in chronic LBP. A pragmatic, randomised trial with a one-year follow-up." BMC Musculoskeletal Disorders **11**: 212-212.
Sparrow, D., D. J. Gottlieb, D. Demolles and R. A. Fielding (2011). "Increases in muscle strength and balance using a resistance training program administered via a telecommunications system in older adults." Journals of Gerontology Series A: Biological Sciences & Medical Sciences **66**(11): 1251-1257.
Spink, M. J., H. B. Menz, M. R. Fotoohabadi, E. Wee, K. B. Landorf, K. D. Hill and S. R. Lord (2011). "Effectiveness of a multifaceted podiatry intervention to prevent falls in community dwelling older people with disabling foot pain: randomised controlled trial." BMJ **342**: d3411.
Stanley, I., J. Miller, M. A. Pinnington, G. Rose and M. Rose (2001). "Uptake of prompt access physiotherapy for new episodes of back pain presenting in primary care." Physiotherapy **87**(2): 60-67.
Stevenson, K., M. Lewis and E. Hay (2004) "Do physiotherapists' attitudes towards evidence-based practice change as a result of an evidence-based educational programme?" Journal of evaluation in clinical practice **10**, 207-217 DOI: 10.1111/j.1365-2753.2003.00479.x.
Taheri, H., R. Mahdavinejad, V. Minasian and A. Karimi (2012) "The effects of an eight-week selected therapeutic exercises course and self-treatment by pamphlet programs on the rate of chronic neck pain and disability among computer users." Journal of Isfahan Medical School **29**.
Tonga, E., A. Daskapan, T. Duger and N. Ozunlu (2012) "Effects of different back health education on fear avoidance belief and occupational performance in chronic low back pain. [Turkish]." Fizyoterapi Rehabilitasyon **23**, 17-25.
Tousignant, M., H. Moffet, P. Boissy, H. Corriveau, F. Cabana and F. Marquis (2011). "A randomized controlled trial of home telerehabilitation for post-knee arthroplasty." Journal of Telemedicine & Telecare **17**(4): 195-198.
Tuzun, S., G. Akyuz, N. Eskiyurt, A. Memis, B. Kuran, A. Icagasioglu, T. Sarpel, F. Ozdemir, N. Ozgirgin, R. Gunaydin, A. Cakci and M. Yurtkuran (2013) "Impact of the training on the compliance and persistence of weekly bisphosphonate treatment in postmenopausal osteoporosis: A randomized controlled study." International journal of medical sciences **10**, 1880-1887 DOI: 10.7150/ijms.5359.
Van Dillen, L. R., B. J. Norton, S. A. Sahrmann, B. A. Evanoff, M. Harris-Hayes, G. W. Holtzman, J. Earley, I. Chou and M. J. Strube (2016). "Efficacy of classification-specific treatment and adherence on outcomes in people with chronic low back pain. A one-year follow-up, prospective, randomized, controlled clinical trial." Manual Therapy **24**: 52-64.
Verhagen, E. A., M. D. Hupperets, C. F. Finch and W. van Mechelen (2011). "The impact of adherence on sports injury prevention effect estimates in randomised controlled trials: looking beyond the CONSORT statement." Journal of Science & Medicine in Sport **14**(4): 287-292.
Walker, J. (2011). "Effective management strategies for osteoarthritis." British Journal of Healthcare Assistants **5**(3): 123-128.

Wand, B. M., C. Bird, J. H. McAuley, C. J. Doré, M. MacDowell and L. H. De Souza (2004). "Early intervention for the management of acute low back pain: a single-blind randomized controlled trial of biopsychosocial education, manual therapy, and exercise." Spine **29**(21): 2350-2356.
Watson, K., A. Wright, N. Morris, J. McMeeken, D. Rivett, F. Blackstock, A. Jones, T. Haines, V. O'Connor, G. Watson, R. Peterson and G. Jull (2012). "Can simulation replace part of clinical time? Two parallel randomised controlled trials." Medical Education **46**(7): 657-667.
Werner, E. L., K. Storheim, I. Lochting, T. Wisloff and M. Grotle (2016). "Cognitive patient education for low back pain in primary care: A cluster randomized controlled trial and cost-effectiveness analysis." Spine **41**(6): 455-462.
Willett, G. M., G. C. Johnson and K. Jones (2011). "The effect of a hybrid continuing education course on outpatient physical therapy for individuals with low back pain." Internet Journal of Allied Health Sciences & Practice **9**(1): 11p.
Williams, A. J., J. Wiggers, K. M. O'Brien, L. Wolfenden, S. Yoong and C. M. Williams (2016). "A telephone-based lifestyle behavioural intervention for overweight or obese patients with low back pain." Obesity Reviews **17**: 156.
Williams, A. M., L. Bloomfield, E. Milthorpe, D. Aspinall, K. Filocamo, T. Wellsmore, N. Manolios, U. W. Jayasinghe and M. F. Harris (2013) "Effectiveness of Moving On: an Australian designed generic self-management program for people with a chronic illness." BMC health services research **13**, 90 DOI: 10.1186/1472-6963-13-90.
Williams, N. H., E. Amoakwa, J. Belcher, R. T. Edwards, H. Hassani, M. Hendry, K. Burton, R. Lewis, K. Hood, J. Jones, P. Bennett, P. Linck, R. D. Neal and C. Wilkinson (2011) "Activity Increase Despite Arthritis (AÏDA): phase II randomised controlled trial of an active management booklet for hip and knee osteoarthritis in primary care." British journal of general practice **61**, e452-458 DOI: 10.3399/bjgp11X588411.
