# Supplementary material for: The effectiveness of interventions designed to increase the uptake of clinical practice guidelines and best practices among musculoskeletal professionals: a systematic review
Source: BMC Health Serv Res. 2018 Jun 8;18:435. doi: 10.1186/s12913-018-3253-0 (PMC5994025; doi:10.1186/s12913-018-3253-0)
Supplement: Supplementary file 3 — The main characteristics of KT interventions and their effectiveness of the professional outcomes. (DOCX 29 kb) [file 12913_2018_3253_MOESM3_ESM.docx]

**The main characteristics of KT interventions and their effectiveness of the professional outcomes**

| **Author (year)** | **KT intervention; groups (number of participants per group)** | **MSK professional; MSK disorder ; duration** | **Primary outcome/s = measure/s; (scale) original unit of results** | **Cohen’s d Significant outcomes: * *P*<.05** |
| --- | --- | --- | --- | --- |
| **Bekkering (2005)** | Multifaceted;  I= Distribution of educational materials (guidelines by mail) + Interactive educational meetings (n=52) C= distribution of educational materials (guidelines by mail) (n=61) | PTs; nonspecific-LBP; Baseline and 4 wks | Adherence to guideline (4 recommendations)= individual patients’ forms recording the treatment completed by the physiotherapist | *d*= 0.4 (0.08 - 0.71)* |
| **Stevenson (2006)** | Multifaceted; I= Educational meetings + local opinion leaders (n=17) C= No intervention (n=13) | Musculoskeletal PTs; LBP; Baseline and 6 mo | Change in PTs’ clinical practice= ‘**discharge summary**’ questionnaires: *Time spent, Importance* | Advice about work situation: *d*= 0.05 (0.67 - 0.77) Advice on return to normal activities: *d*= 0.05 (0.67 - 0.77) Advice to increase activity level: *d*= 0.41 (-0.32 - 1.14) Encourage early return to work: *d*= 0.26 (-0.47 - 0.99) Encourage to do activities themselves: *d*= -0.66 (-1.40 - 0.08) Change attitudes/beliefs about pain: *d*= 0.53 (-0.20 - 1.26) |
| **Rebbeck (2006)** | Multifaceted; I= (distribution of educational materials; Interactive educational meeting; local opinion leaders, outreach visit) (n= 14) C= (distribution of educational materials (Guidelines by mail)) (n= 13) | PTs; Whiplash; Baseline, 1.5mo. 3mo, 6 mo, and 12mo | PT knowledge about the guidelines= a custom-made questionnaire (0-28) | *d*= 2.151 (1.2 - 3.1)* |
| **Bussières (2010)** | Multifaceted; I= (Educational meeting + reminder) (n= 80) C= (No intervention) (n= 80) | DCs; Spinal Disorders; Baseline, 6–8 wks, 14-16 wks | Appropriate use of the diagnostic imaging: measured using rate of appropriate responses for the use of diagnostic imaging were three questionnaires, each consisting of 10 different spine case scenarios | *d*= 0.05 (-0.26 - 0.36) |
| **Evans (2010)** | Single I= (Educational materials) (n= 876) C= (No intervention) (n= 882) | DCs, osteopaths, and PTs; Acute LBP; baseline and at 6 mo | Professional behavior: measured using 3 “quality indicators” (activity, work, and bed-rest) relating to a vignette of a patient with LBP - Dichotomous | Activity: *d*= 0.14 (0.05 - 0.23)* Work: *d*= 0.16 (0.07 - 0.25)*  Bed-rest: *d*= 0.15 (0.06 - 0.24) |
| **Peter (2013)** | Single I= (Interactive educational meetings) (n= 124) C= (conventional educational meetings) (n= 124) | PTs; OA for hip and knee; before the educational course, immediately afterwards, and 3 months thereafter | Guideline Adherence: Quality Indicators for Physical Therapy in Hip and Knee Osteoarthritis [QIP-HKOA] (scale 0-72) | *d*= 0.28 (0.02 - 0.53)* |
| **Peter (2015)** | Single I= (Interactive educational meetings) (n = 160) C= No intervention (n = 159) | PTs; OA for hip and knee; 1 week before the interactive course, immediately after, and 3 months thereafter | **Adherence** to guideline (score range 0–24) | *d*= 0.23 (0.01 - 0.45)* |
| **van Dulmen (2014)** | Multifaceted; I= (educational meetings, written materials, other (peer-assessment)) (n= 44) C= (educational meetings, written materials) (n= 34) | PTs; LBP; baseline and at 6 mo | Knowledge and guideline-consistent reasoning, measured with 12 performance indicators using 4 vignettes with speciﬁc guideline-related patient proﬁles. For each participant, the total score was calculated by adding up the percentage scores (0 –100) per vignette, divided by 4. | *d*= 0.824 (-0.23 - 1.88)* |
| **Murray (2015)** | Multifaceted; I= multicomponent (educational meetings, reminders) (n=12) C= single No intervention) (n=12) | PTs; chronic LBP; Varies according to patient-physiotherapist audio recorded 16.7± 6.9 weeks after the end of the CONNECT intervention | Verbal communication between each physiotherapist and a patient was recorded on an audiotape, and independent, blinded raters used the Health Care Climate Questionnaire (HCCQ) to assess physiotherapists’ needs-supportive behavior - 6-item: to assess PTs’ needs-supportive communication. Using a computer-based algorithm, an independent researcher randomly assigned audio recordings to the 3 raters. Raters each listened to 12 recordings and used the HCCQ.  The scale includes 7-point Likert scales [1 (not true at all), 7 (very true)]. | *d*=2.27 (1.24 - 3.29)* |
| **Chipchase (2016)** | Multifaceted; I= multicomponent (educational meetings, reminders) (n=12) C= single (educational meeting) (n=11) | PTs; Neck pain; prior to the ﬁrst weekend workshop and 2 months after the workshop | Self-reported PT practice behaviour and conﬁdence: measured using a semi-structured questionnaire containing closed (Likert-type responses) and open questions. The questionnaire had two sections:  1. questions related to practitioner conﬁdence in the assessment of cervical motor and sensorimotor function, as well as the prescription and progression of exercise in the management of patients with neck pain (Category codes were: 1 = not conﬁdent; 2 = somewhat conﬁdent; 3 = conﬁdent; 4 = very conﬁdent).  2. Information on their usual management strategies for patients with neck pain (Category codes were: 1 = not at all; 2 = some of the time; 3 = most of the time; 4 = all of the time). | **Practice behaviour section 1**:  Assessment: *d*=0.2 (-0.62 - 1.02) Clinical reasoning: *d*= 0.41 (-0.42 - 1.24) Progression of exercises: *d*= 0.2 (-0.62 - 1.02). **Practice behaviour section 2**: there is no one summary score for the questionnaire. However, the authors mentioned that: "there was no change in practitioners' reports of the frequency with which they used any of these management strategies (p > 0.05)". |

I: Intervention group; C: Control group; Wk: week; mo: months; 95% CI; 95% Confidence interval; *d*: Effect size; PT: Physical therapist; LBP: Low back pain; DC: Doctor of chiropractors; OA: Osteoarthritis
