# Supplementary material for: The effectiveness of interventions designed to increase the uptake of clinical practice guidelines and best practices among musculoskeletal professionals: a systematic review
Source: BMC Health Serv Res. 2018 Jun 8;18:435. doi: 10.1186/s12913-018-3253-0 (PMC5994025; doi:10.1186/s12913-018-3253-0)
Supplement: Supplementary file 4 — The main characteristics of KT interventions and their effectiveness on patient outcomes. (DOCX 18 kb) [file 12913_2018_3253_MOESM4_ESM.docx]

**The main characteristics of KT interventions and their effectiveness on patient outcomes**

| **Author (year)** | **KT intervention; groups (number of participants per group)** | **MSK professional; MSK disorder; duration** | **Primary outcome/s = measure/s; (scale) original unit of results** | **Cohen’s d Significant outcomes: * *P*<.05** |
| --- | --- | --- | --- | --- |
| **Bekkering (2005)** | Multifaceted;  I= Distribution of educational materials (guidelines by mail) + Interactive educational meetings (n=247) C= Distribution of educational materials (guidelines by mail) (n=253) | PTs; nonspecific-LBP; Baseline, 6 wks, 12 wks, 26 wks, 52 wks | Disability= Quebec Back Pain Disability Scale [QBPDS]; (0–100) pain= 11-point numeric rating scale [NRS]; (0–10) | QBPDS *d*=0.28 (0.10-0.45) NRS *d*= 0.31 (0.13-0.49) |
| **Rebbeck (2006)** | Multifaceted; I= (distribution of educational materials; educational meeting; local opinion leaders, outreach visit) (n= 67) C= (distribution of educational materials (Guidelines by mail)) (n= 26) | PTs; Whiplash; Baseline, 1.5mo. 3mo, 6 mo, and 12mo | Disability= Functional Rating Index (FRI); (0-40) disability due to acute whiplash = adapted version of the 7-item Core Outcome Measure for neck pain; (5 - 25) | FRI *d*= 0.06 (-0.39 - 0.51) Core outcome measure *d*= -0.12 (-0.33 - 0.57) |
| **Cleland (2009)** | Multifaceted; I= (Educational meetings + Educational outreach visit) (n= 283) C= (No intervention) (n= 228) | PTs; neck pain; Baseline and 12 mo | Disability= Neck Disability Index (NDI) - (%) Pain= numeric pain rating scale (NPRS) - (0 - 10) | Disability *d*= 0.27 (0.09 - 0.45)* Pain *d*= 0.16 (-0.01 - 0.33) |
| **Chipchase (2016)** | Multifaceted; I= multicomponent (educational meetings, reminders) (n= 62) C= single (educational meeting) (n= 53) | PTs; Neck pain; 2 mo before and 2 months after the workshop | Neck Disability Index (NDI) [0-50] | *d*= 0.11 (-0.25 - 0.48) |

I: Intervention group; C: Control group; Wk: week; mo: months; 95% CI; 95% Confidence interval; *d*: Effect size; PT: Physical therapist; LBP: Low back pain
