# Supplementary material for: The effectiveness of interventions designed to increase the uptake of clinical practice guidelines and best practices among musculoskeletal professionals: a systematic review
Source: BMC Health Serv Res. 2018 Jun 8;18:435. doi: 10.1186/s12913-018-3253-0 (PMC5994025; doi:10.1186/s12913-018-3253-0)
Supplement: Supplementary file 5 — Elements of KT interventions and use of theoretical frameworks, models or theories. (DOCX 31 kb) [file 12913_2018_3253_MOESM5_ESM.docx]

**Elements of KT interventions and use of theoretical frameworks, models or theories**

| **Author (year)** | **Targeted MSK professionals** | **EPOC professional intervention classification** | **Type of targeted behaviour** | **Evidence source** | **Format** | **Delivered by** | **Frequency** | **Duration of each session** | **Theoretical framework** | **Manual availability** | **Training of providers** |
| --- | --- | --- | --- | --- | --- | --- | --- | --- | --- | --- | --- |
| **Bekkering (2005)** | PTs | Professional - Distribution of educational materials + Interactive educational meetings | General management of a problem | Dutch physical therapy guidelines for low back pain | Oral communication, written material, role-playing with actors, reminder | Health professional, researcher, self-administered | 2 | 2.5h | Changing behaviour model | No | NR |
| **Stevenson (2006)** | MSK PTs | Professional - Educational meetings + local opinion leaders | General management of a problem | Best evidence about ‘Psychosocial Yellow Flags’ | Oral communication | Health professional (PTs) | 2 | 2.5h | Opinion leaders’ theory | No | NR |
| **Rebbeck (2006)** | PTs | Professional - Interactive educational meeting + Educational outreach  visits + Distribution of educational materials | General management  of a problem | Clinical practice guidelines for the management of acute whiplash | Oral communication, written material, practical training | Health professional (PTs) | 2 | 8h Outreach visit= 2h | No | No | NR |
| **Cleland (2009)** | PTs | Professional - Educational meetings + Educational outreach visit | General management of a problem | Best evidence about classiﬁcation system for the management of neck pain | Oral communication, written material, practical training | Health professional (PTs) | 2 meeting and 1 outreach visit | 1.5h/meeting and 1hr for the visit | No | No | NR, however, PTs were Orthopaedic Certiﬁed Specialists and had fellowship status in the American Academy of Orthopaedic Manual Therapy |
| **Bussières (2010)** | DCs | Professional - Educational meeting + Reminder | Diagnosis | Diagnostic Imaging Guidelines- recommendations for spine disorders | Oral communication, reminder | Health professional (specialist DCs) | 1 | 1.5h | No | No | NR, however, the DCs were specialists (one in clinical sciences and one in radiology) |
| **Evans (2010)** | DCs, osteopaths, and PTs | Professional - Distribution of educational materials | General management of a problem | UK primary care and occupational CPG for acute LBP | Written material | Self-administered (mailing) | NR | NR | No | No | NR |
| **Peter (2013)** | PTs | Professional - Educational meetings | General management of a problem | Dutch physiotherapy clinical practice guideline for hip and knee | Oral communication, written material, practical training | Health professional (expert PT and PT teachers) | 1 | 3h | first 3 levels of Kirkpatrick model | No | Yes, 1.5 h |
| **Peter (2015)** | PTs | Professional - Educational meetings | General management of a problem | Dutch physiotherapy clinical practice guideline for hip and knee | Oral communication, written material, practical training | Health professional (expert PT and PT teachers) | 1 | 3h | first 3 levels of Kirkpatrick model | Yes | Yes, 1.5 h |
| **van Dulmen (2014)** | PTs | Professional - educational meetings + other (peer-assessment) | General management of a problem | The updated Dutch LBP guideline for physical therapists | Oral communication, written material, clinical reasoning, peer assessment and the case-based discussions | Health professional (expert assessor PT) | 4 | 2 h | No | No | Peer-assessment coached by an expert assessor, a physical therapist with expertise in LBP and an experienced teacher. |
| **Murray (2015)** | PTs | Professional - Educational meeting + Reminder | Professional-patient communication | Evidence-based physiotherapy care for CLBP, including recommendations to include PA as part of home-based rehabilitation…..based on current evidence-based clinical guidelines for CLBP. | Oral communication, Video recordings of simulated initial treatment sessions, active role-play and group discussion | Workshop leader | 2 | 4h | Self-Determination Theory, 5A’s Framework of Behavior Change (well-explained) | No | No training mentioned for the workshop leader |
| **Chipchase (2016)** | PTs | Professional - educational meetings + follow-ups | General management of a problem | The two-day workshop provided an evidence-based approach towards the diagnosis and management of neck disorders, with an emphasis on multimodal interventions inclusive of advice, education, exercise and manual therapy. The course especially promoted a research-informed therapeutic exercise program within the multimodal program that has been shown to be efﬁcacious for persons with neck disorders in clinical trials (Jull et al., 2002, 2007). | Oral communication, practical training | Health professional (MSK Expert PT and experienced educator) | 2 | 12.5h | No | No | A specialist MSK PT, experienced researcher and Fellow of the Australian College of PT. An experienced educator familiar with the workshop material assisted the lead instructor. |
| MSK: Musculoskeletal; PT: Physical therapists; DCs: Doctor of chiropractic; NR: Not reported; CLBP: Chronic low back pain. | | | | | | | | | | | |
